# Supplementary material for: Sampling bias and model choice in continuous phylogeography: Getting lost on a random walk
Source: PLoS Comput Biol. 2021 Jan 6;17(1):e1008561. doi: 10.1371/journal.pcbi.1008561 (PMC7815209; doi:10.1371/journal.pcbi.1008561)
Supplement: S1 Text — The Supplement contains additional information regarding the methods used. (PDF) [file pcbi.1008561.s001.pdf]

Sampling bias and model choice in continuous phylogeography: getting lost on a random walk - Supplementary Text S1

Antanas Kalkauskas[1, ‡, ¤], Umberto Perron[1, ‡], Yuxuan Sun[1], Nick Goldman[1], Guy Baele[2], Stephane Guindon[3], Nicola De Maio[1,*]

1 European Molecular Biology Laboratory, European Bioinformatics Institute, Wellcome Genome Campus, Hinxton, Cambridgeshire, CB10 1SD, UK
2 Department of Microbiology, Immunology and Transplantation, Rega Institute, KU Leuven, Herestraat 49, 3000 Leuven, BE
3 Department of Computer Science, LIRMM, CNRS and Université de Montpellier, Montpellier, FR

‡ These authors contributed equally to this work.
¤ Current address: Sensmetry, UAB, J. Jasinskio st. 16A, 03163 Vilnius, Lithuania.
* demaio@ebi.ac.uk

1 Supplement

Estimates of diffusion rate σ^2

Defining σ^2 for the ΛFV in *discsim*

Here we want to relate the dispersion rate of lineages under the ΛFV simulation model in *discsim* with the diffusion rate inferred by PhyREX and the BMP in BEAST. To do this, we consider what is the dispersion rate of lineages in *discsim* given certain model parameter values, and in particular we focus on the limit of frequent but small events, so that lineages migrate approximately in a Brownian motion over short times (see [1]). Given λ the rate of events in the disc-based ΛFV, μ the death probability, r the radius of event discs, and assuming the considered space A is a torus with both dimensions of size L (see main text for a description of the ΛFV), then the rate of events overlapping with the location of a certain individual is:

$$\frac{\lambda \pi r^2}{L^2}.$$

When an individual is covered by the disc D of an event, the probability that it dies is μ. When a new individual is born at location (x_2, y_2), the mean square distance of one-event displacement from its parent at (x_1, y_1) is the mean square distance between two points chosen uniformly at random in the disc; this is r^2, as can be seen from the following (see also [2]):

$$\int_D \left(\int_D ((x_1 - x_2)^2 + (y_1 - y_2)^2) \frac{1}{\pi r^2} dx_1 dy_1 \right) \frac{1}{\pi r^2} dx_2 dy_2$$

$$= \int_D \left(\int_D (x_1^2 + x_2^2 - 2x_1 x_2 + y_1^2 - 2y_1 y_2 + y_2^2) \frac{1}{\pi r^2} dx_1 dy_1 \right) \frac{1}{\pi r^2} dx_2 dy_2$$

$$= \int_D \left(\int_D (x_1^2 + x_2^2 + y_1^2 + y_2^2) \frac{1}{\pi r^2} dx_1 dy_1 \right) \frac{1}{\pi r^2} dx_2 dy_2$$

$$= \int_0^{2\pi}\int_0^r\int_0^{2\pi}\int_0^r (\rho_1^2+\rho_2^2)\frac{1}{\pi^2 r^4}\rho_1\rho_2 d\rho_1 d\theta_1 d\rho_2 d\theta_2$$

$$= \frac{4}{r^4}\int_0^r\int_0^r (\rho_1^2+\rho_2^2)\rho_1\rho_2 d\rho_1 d\rho_2 = \frac{4}{r^4}\int_0^r \left(\frac{r^4}{4}\rho_2 + \rho_2^3\frac{r^2}{2}\right) d\rho_2 = \frac{4}{r^4}\left(\frac{r^6}{8}+\frac{r^6}{8}\right) = r^2$$

since, without loss of generality, we can assume that D is centred at 0 and observing that terms such as $2x_1x_2$ integrate to 0.

So, over a very short time t, the mean square displacement of a lineage is

$$t\frac{\lambda\mu\pi r^2}{L^2}r^2 = t\frac{\lambda\mu\pi r^4}{L^2}$$

and the diffusion rate per dimension is

$$\sigma^2 = \frac{\lambda\mu\pi r^4}{2L^2}.$$

To enforce $\sigma^2 = 1$ we therefore simulate under the condition $\lambda = \frac{2L^2}{\mu\pi r^4}$, and in particular with $L = 100$, $r = 0.1$, and $\mu = 0.1$.

Estimating σ^2 from the ΛFV in PhyREX

A theoretical estimate of σ^2 from the PhyREX inference is obtained similarly as

$$\sigma^2 = \frac{4\lambda\mu\pi\theta^4}{L^2} \qquad (1)$$

following [3], where θ, similarly to r, measures the spatial size of events. Throughout the manuscript, we use this classical measure of σ^2. However, as this is an approximation assuming a limit of a Brownian motion, we also test alternative statistics below, which however seem overall less reliable estimates.

In one of the statistics, “dispersion from the root”, we consider the average squared Euclidean distance from the current root location (at the current MCMC step) and the tip locations, and divide this by twice the time distance between the root and the tips (the tips are all assumed collected at the same time). Because lineages are inferred to travel several times across the considered space before coalescing, the dispersion from the root statistic would usually not represent the instantaneous dispersion rate of lineages well; in fact, we see in S14 Fig that this measure severely underestimates the diffusion rate from *discsim* simulations.

As another alternative we also consider the “dispersion across short distance from the tips”, which is the sum of the squared Euclidean distances between each tip and its location after (backward in time) its first event affecting its location, divided by the sum of the times for each tip to each such event. This “dispersion near the tips statistic” better summarizes the short-term dispersion of lineages in the PhyREX model; however, this statistic seems to both underestimate the diffusion rate in *discsim* simulations (S14 Fig) and to overestimate it in BMP simulations (S12 Fig).

Finally, we considered as a statistic the “dispersion across long distance from the tips”, which is half the average square Euclidean distance of the tips from their ancestral position one time unit in the past. This statistic seems to overestimate the diffusion rate in BMP simulations (S12 Fig) while giving overall unreliable estimates in *discsim* simulations (S14 Fig).

All four estimates of σ^2 mentioned above (the theoretical one, the dispersion from the root, dispersion across short distance from the tips, and the dispersion across long distance from the tips) have been included in PhyREX and are now part of its output.

σ^2 for the BMP

For the BMP in our simulations we used identity diffusion and precision matrices, which leads, over a short time t, to a mean square displacement of $2t$ and so to $\sigma^2 = 1$.

References

1. Barton NH, Kelleher J, Etheridge AM. A new model for extinction and recolonization in two dimensions: quantifying phylogeography. Evolution: International journal of organic evolution. 2010;64(9):2701–2715.

2. García-Pelayo R. Distribution of distance in the spheroid. Journal of physics A: mathematical and general. 2005;38(16):3475.

3. Guindon S, Guo H, Welch D. Demographic inference under the coalescent in a spatial continuum. Theoretical population biology. 2016;111:43–50.

4. Pybus OG, Suchard MA, Lemey P, Bernardin FJ, Rambaut A, Crawford FW, et al. Unifying the spatial epidemiology and molecular evolution of emerging epidemics. Proceedings of the national academy of sciences. 2012;109(37):15066–15071.

5. Faria NR, Kraemer MU, Hill S, De Jesus JG, Aguiar R, Iani FC, et al. Genomic and epidemiological monitoring of yellow fever virus transmission potential. Science. 2018;361(6405):894–899.
