# Supplementary figures and images for: Sampling bias and model choice in continuous phylogeography: Getting lost on a random walk
Source: PLoS Comput Biol. 2021 Jan 6;17(1):e1008561. doi: 10.1371/journal.pcbi.1008561 (PMC7815209; doi:10.1371/journal.pcbi.1008561)

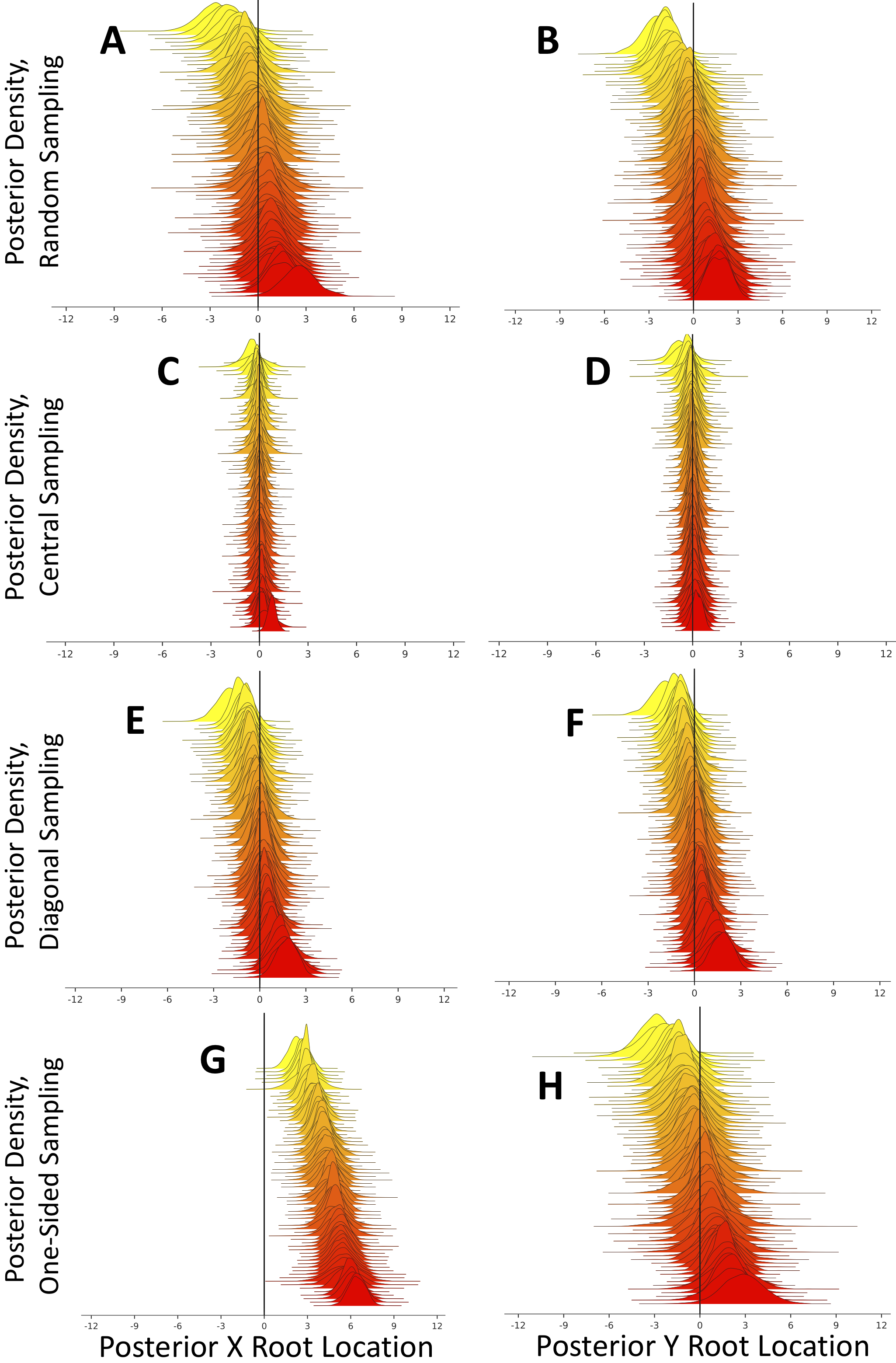

Supplement: S1 Fig — Here a BMP model was used both for simulation and inference. Plots show inferred posterior distributions for the X dimension position of the tree root (plots A,C,E,G), and its Y dimension position (plots B,D,F,H). In each plot, the 100 distributions represent 100 independent replicates, and are vertically sorted based on the posterior median. Vertical black lines show the true, simulated values (in this case always 0). Plots A,B are from simulations with non-biased samples, plots C,D with “Central” biased samples, plots E,F with “Diagonal” biased samples, and plots G,H with “One-sided” sampling bias. Since in many cases the MRCA of the collected samples is not the root of the whole simulated phylogeny (which was simulated at location (0, 0)), in each simulation all locations are translated (in mathematical sense) so that the true simulated sample MRCA is always at (0, 0). (PNG) [file pcbi.1008561.s002.png]

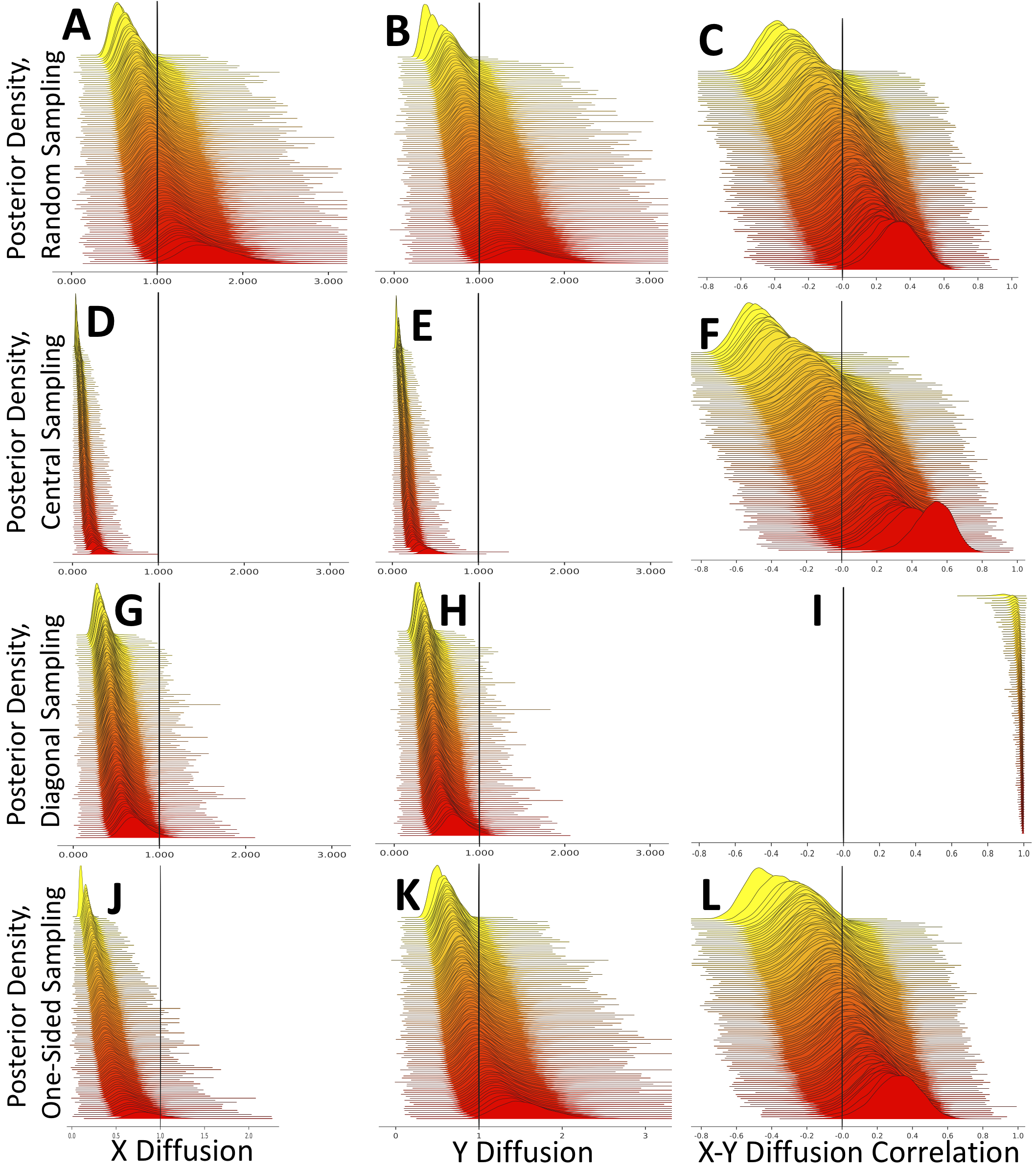

Supplement: S2 Fig — Here a BMP model was used both for simulation and for inference. Plots show inferred posterior distributions for the diffusion rate in the X dimension (plots A,D,G,J), in the Y dimension (plots B,E,H,K), and for the correlation between the diffusion in the two dimensions (plots C,F,I,L). In each plot, the 100 distributions represent 100 independent replicates, and are vertically sorted based on the posterior median. Vertical black lines show the true, simulated values (in this case 1 for rates and 0 for the correlation). Plots A-C are from simulations with non-biased samples, plots D-F with “Central” biased samples, plots G-I with “Diagonal” biased samples, and plots J-L with “One-sided” sampling bias. (PNG) [file pcbi.1008561.s003.png]

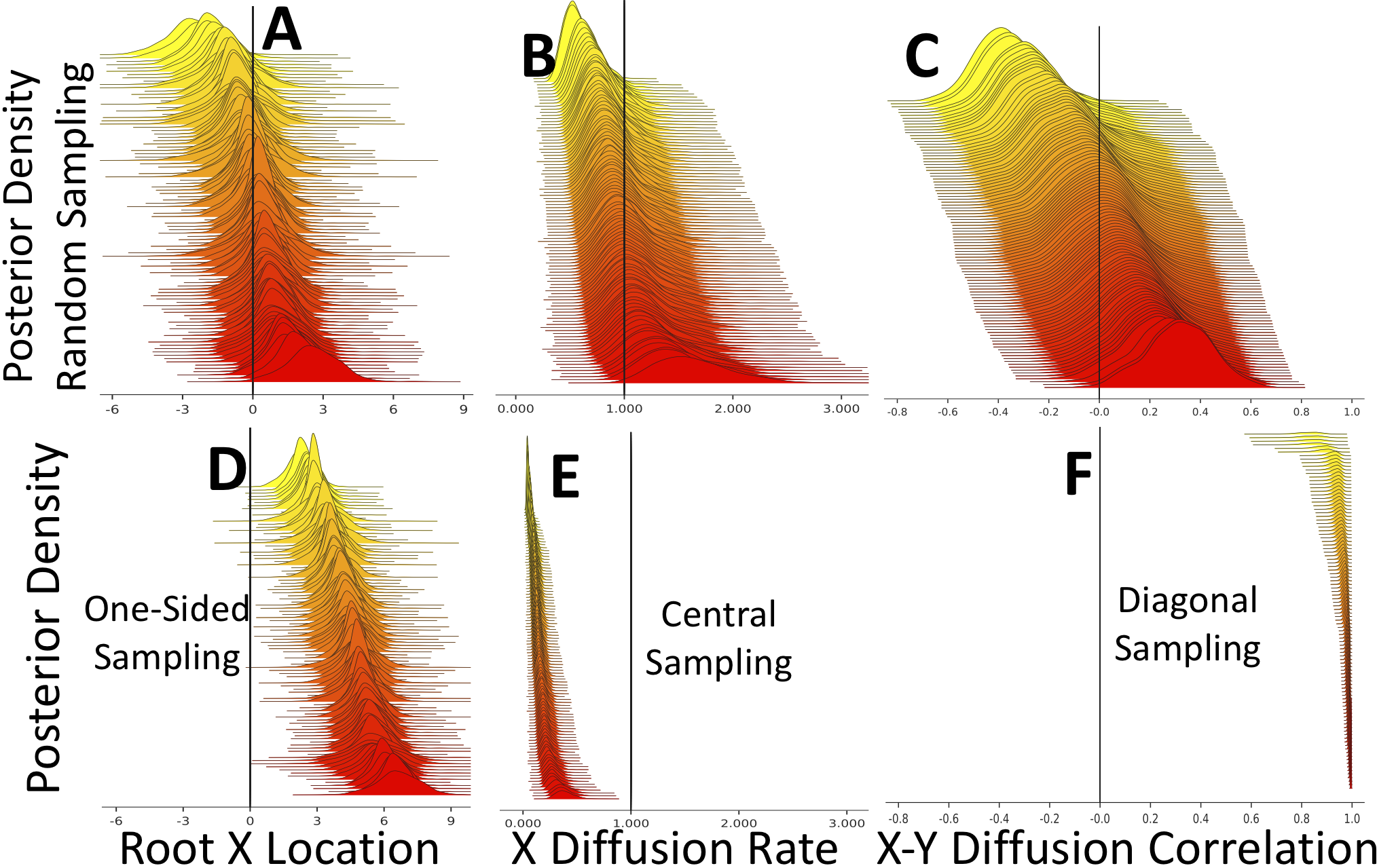

Supplement: S3 Fig — Here a BMP model was used both for simulation and inference, and the phylogenetic tree is assumed to be known without uncertainty. Plots show inferred posterior distributions for the X dimension position of the tree root (plots A,D), the diffusion rate along the X dimension (plots B,E), and the correlation between the diffusion in the two dimensions (plots C,F). In each plot, the 100 distributions represent 100 independent replicates, and are vertically sorted based on the posterior median. Plots A-C are from simulations with non-biased samples. Plots D,E,F are respectively with “One-sided” sampling bias, “Central” sampling bias, and “Diagonal” sampling bias. (PNG) [file pcbi.1008561.s004.png]

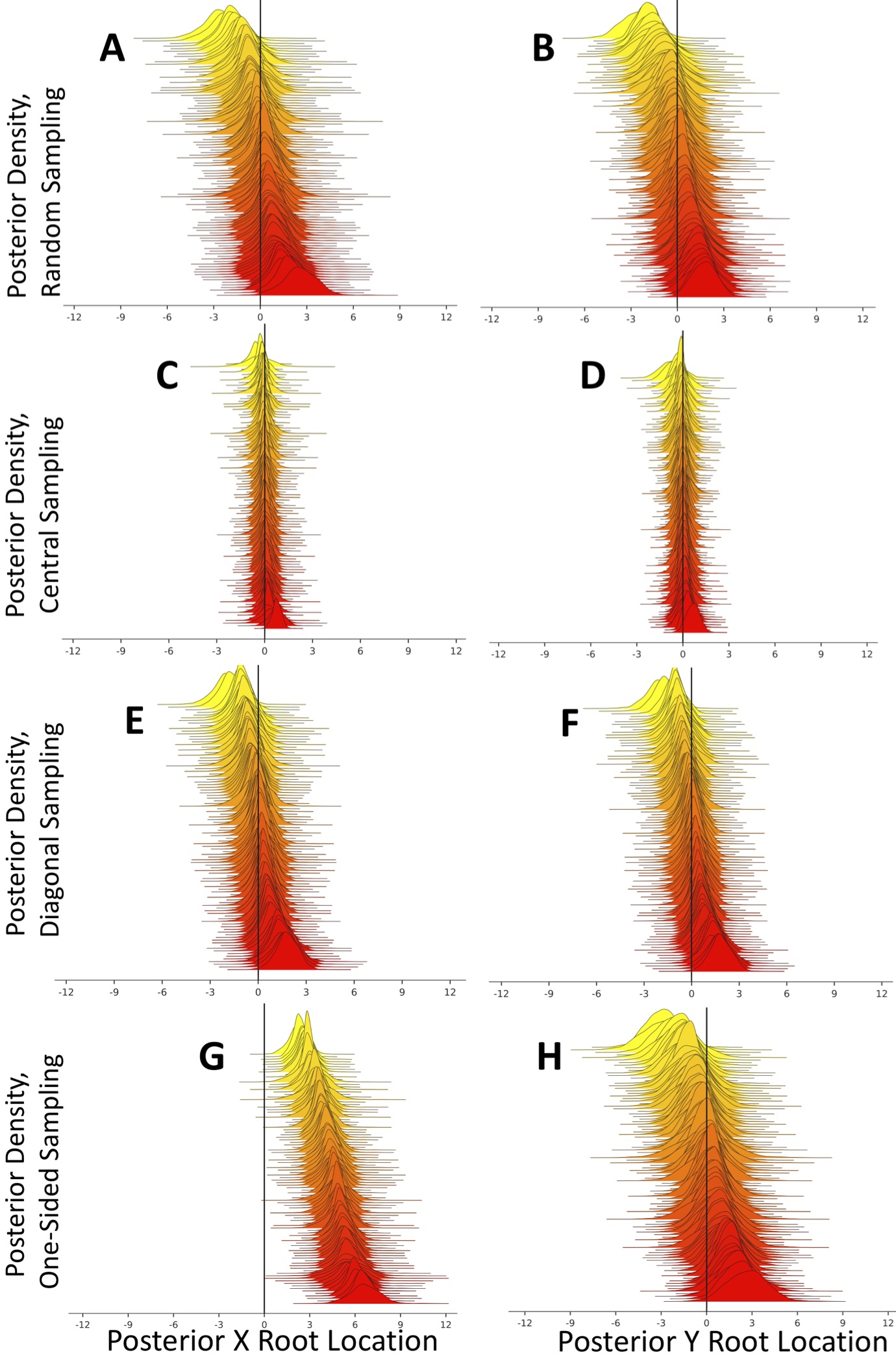

Supplement: S4 Fig — Similarly to S1 Fig, here we show BMP inference of root locations under BMP simulations, but this time the phylogenetic tree is assumed to be known without uncertainty. (JPG) [file pcbi.1008561.s005.jpg]

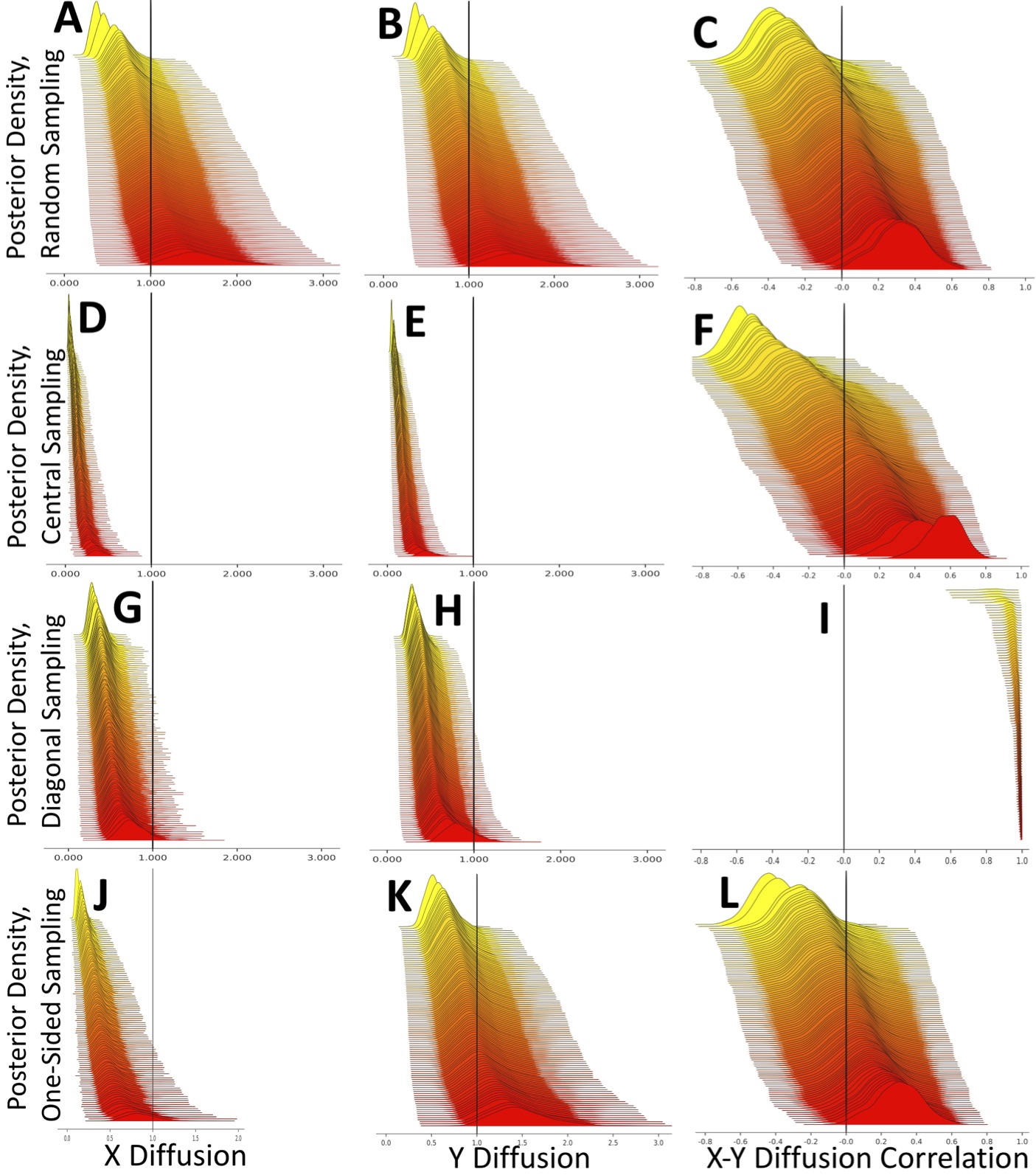

Supplement: S5 Fig — Similarly to S2 Fig, here we show BMP inference of diffusion parameters under BMP simulations, but this time the phylogenetic tree is assumed to be known without uncertainty. (JPG) [file pcbi.1008561.s006.jpg]

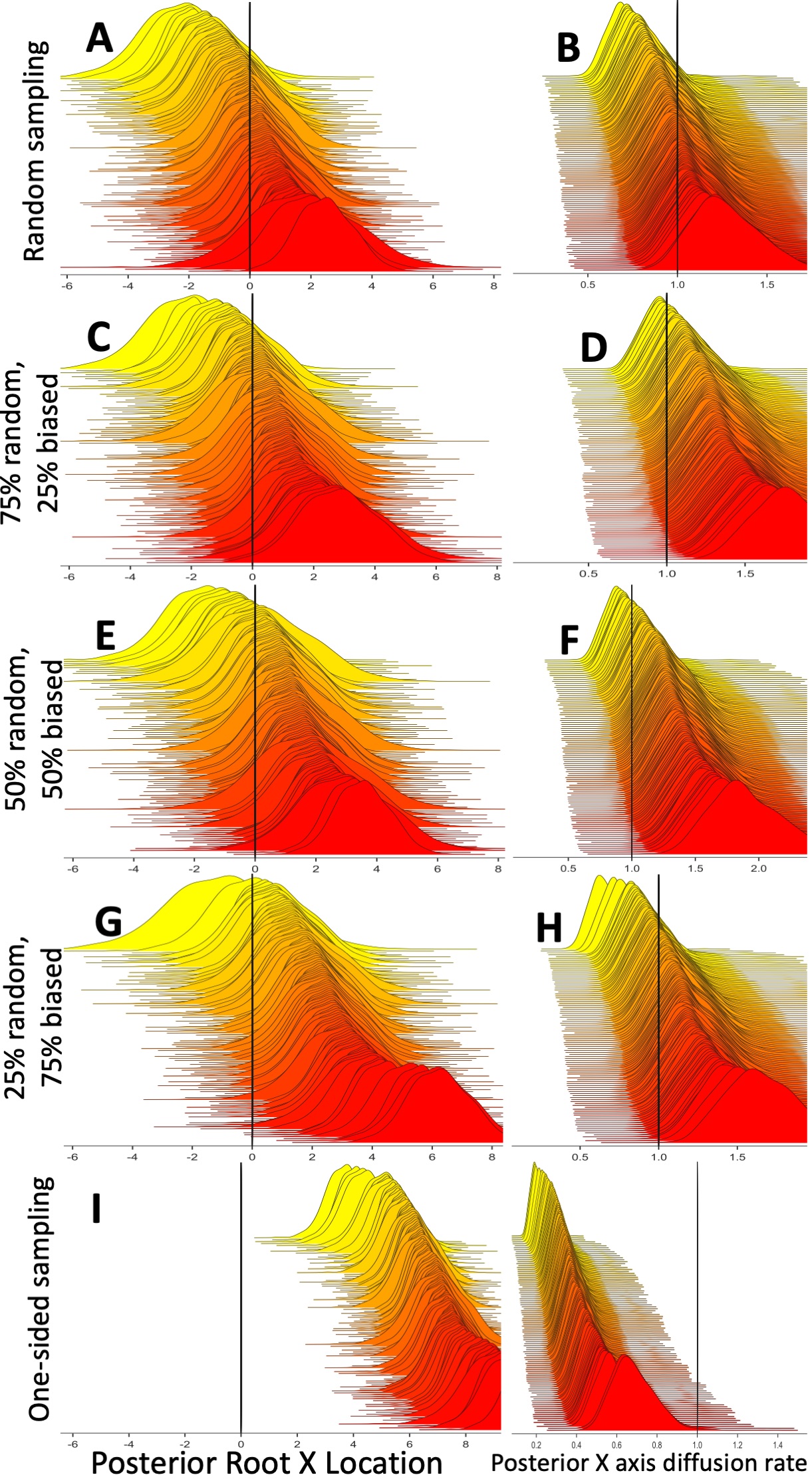

Supplement: S6 Fig — A BMP model was used both for simulation of 10,000 cases and inference from 100 samples. Plots show inferred posterior distributions for the X dimension position of the tree root (plots A,C,E,G,I) and the diffusion rate along the X dimension (plots B,D,F,H,J). In each plot, the 100 distributions represent 100 independent replicates, and are vertically sorted based on the posterior median. Plots A,B are from simulations with non-biased samples, while plots I,J are from simulations where all samples are biased. The other plots show intermediate levels of sampling bias, where 25% (plots C,D), 50% (plots E,F), and 75% (plots G,H) of samples are respectively collected at the positive extreme end of the X axis range, while the remaining samples are randomly selected. (JPG) [file pcbi.1008561.s007.jpg]

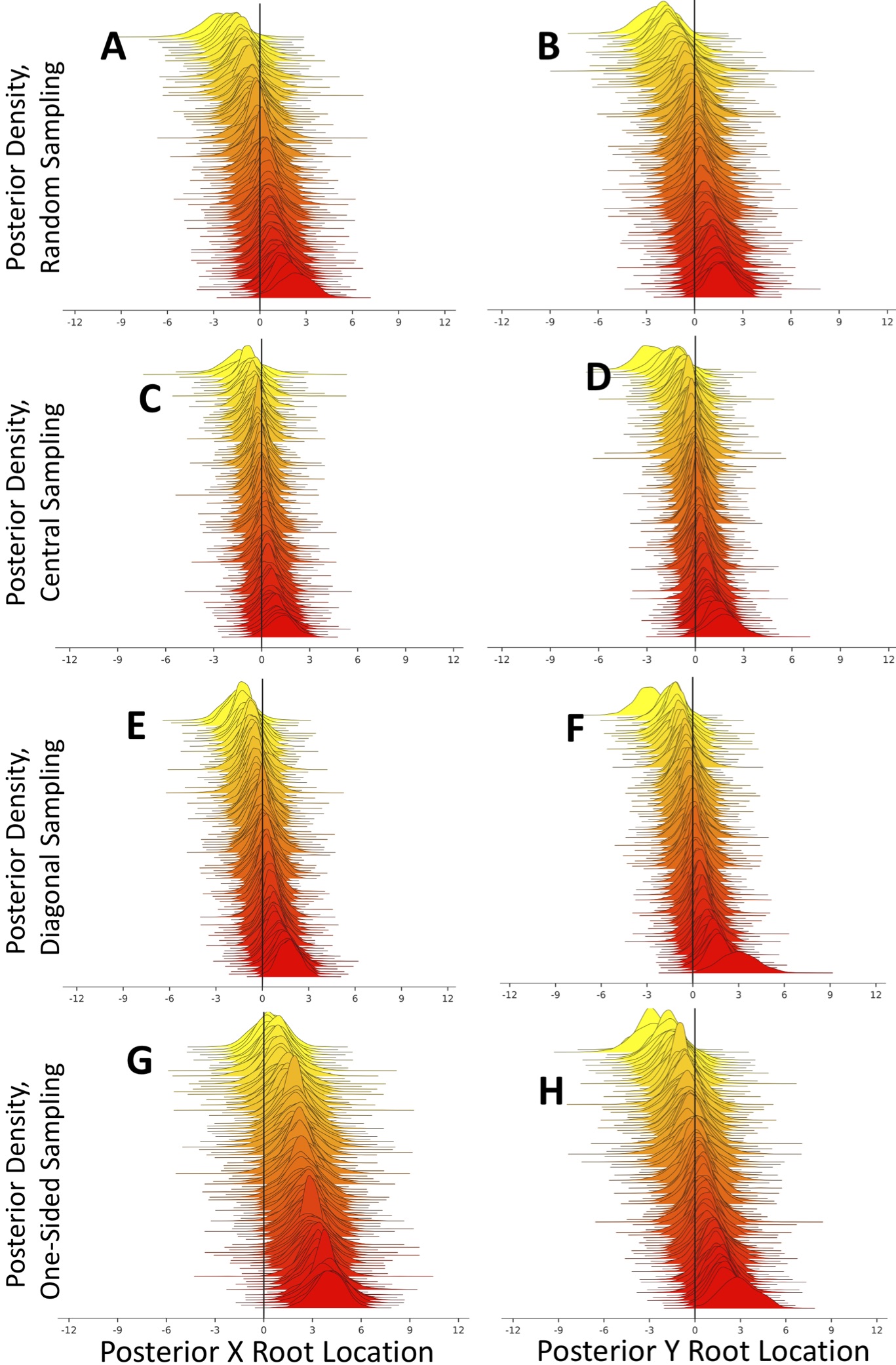

Supplement: S7 Fig — Similarly to S1 Fig, here we show BMP inference of root locations under BMP simulations, but this time we include 50 extra sequence-free samples (without genetic sequence but with correct date and sampling location). (JPG) [file pcbi.1008561.s008.jpg]

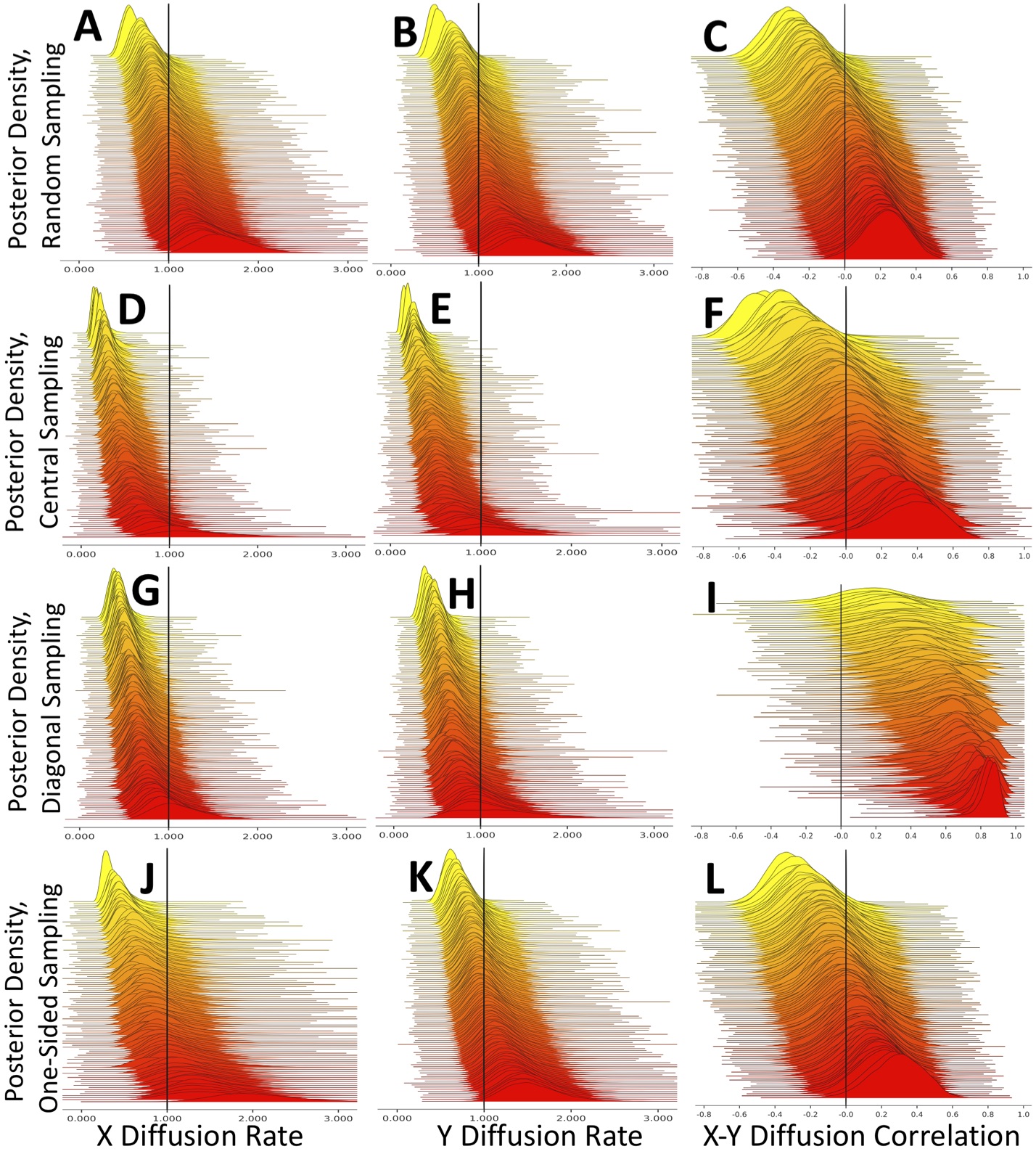

Supplement: S8 Fig — Similarly to S2 Fig, here we show BMP inference of diffusion parameters under BMP simulations, but this time we include 50 extra sequence-free samples (without genetic sequence but with correct date and sampling location). (JPG) [file pcbi.1008561.s009.jpg]

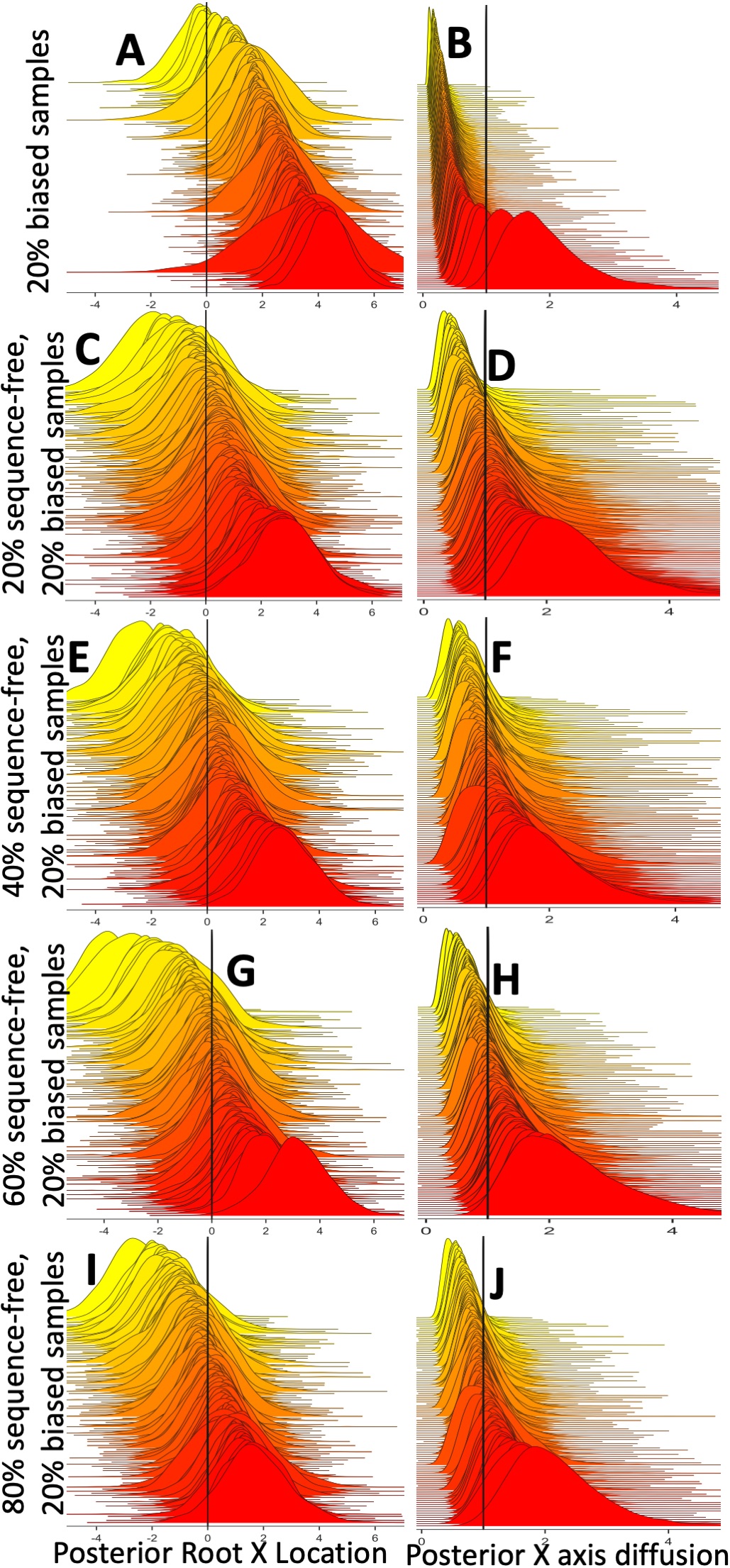

Supplement: S9 Fig — Here we simulate under a BMP model and “One-sided” sampling bias, but only simulate 100 cases and sample 20 of them. A-B Show inference of root X location and diffusion rate in the X dimension respectively. C, E, G, I show inference of root X location after adding some (respectively 20, 40, 60, and 80) of the 80 non-sampled cases to the analysis as sequence-free samples. D, F, H, J show inference of diffusion rate in the X dimension in the same scenarios. (JPG) [file pcbi.1008561.s010.jpg]

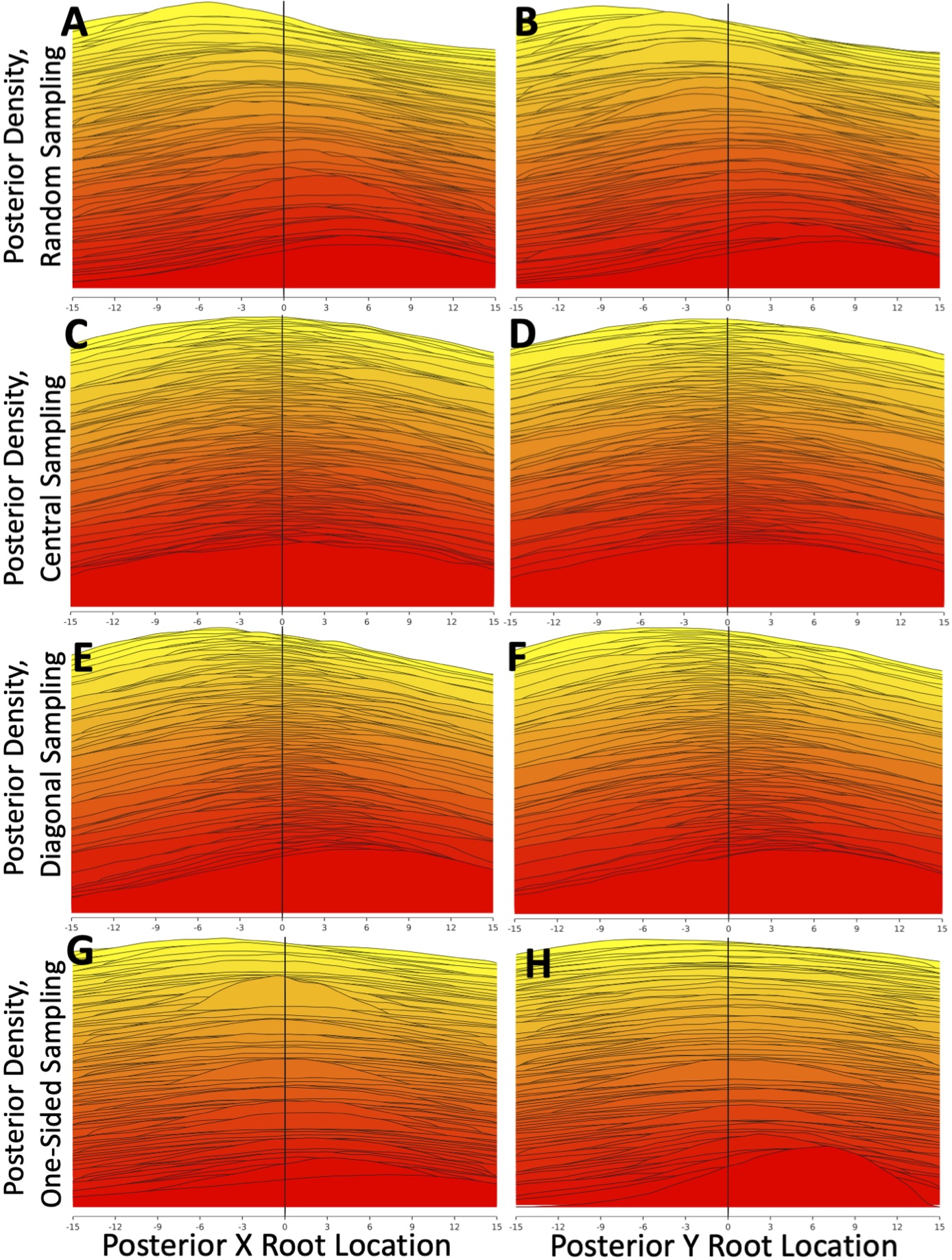

Supplement: S10 Fig — Similarly to S1 Fig, here we show inference of root locations under BMP simulations, but this time inference is performed under the ΛFV model implemented in PhyREX. (JPG) [file pcbi.1008561.s011.jpg]

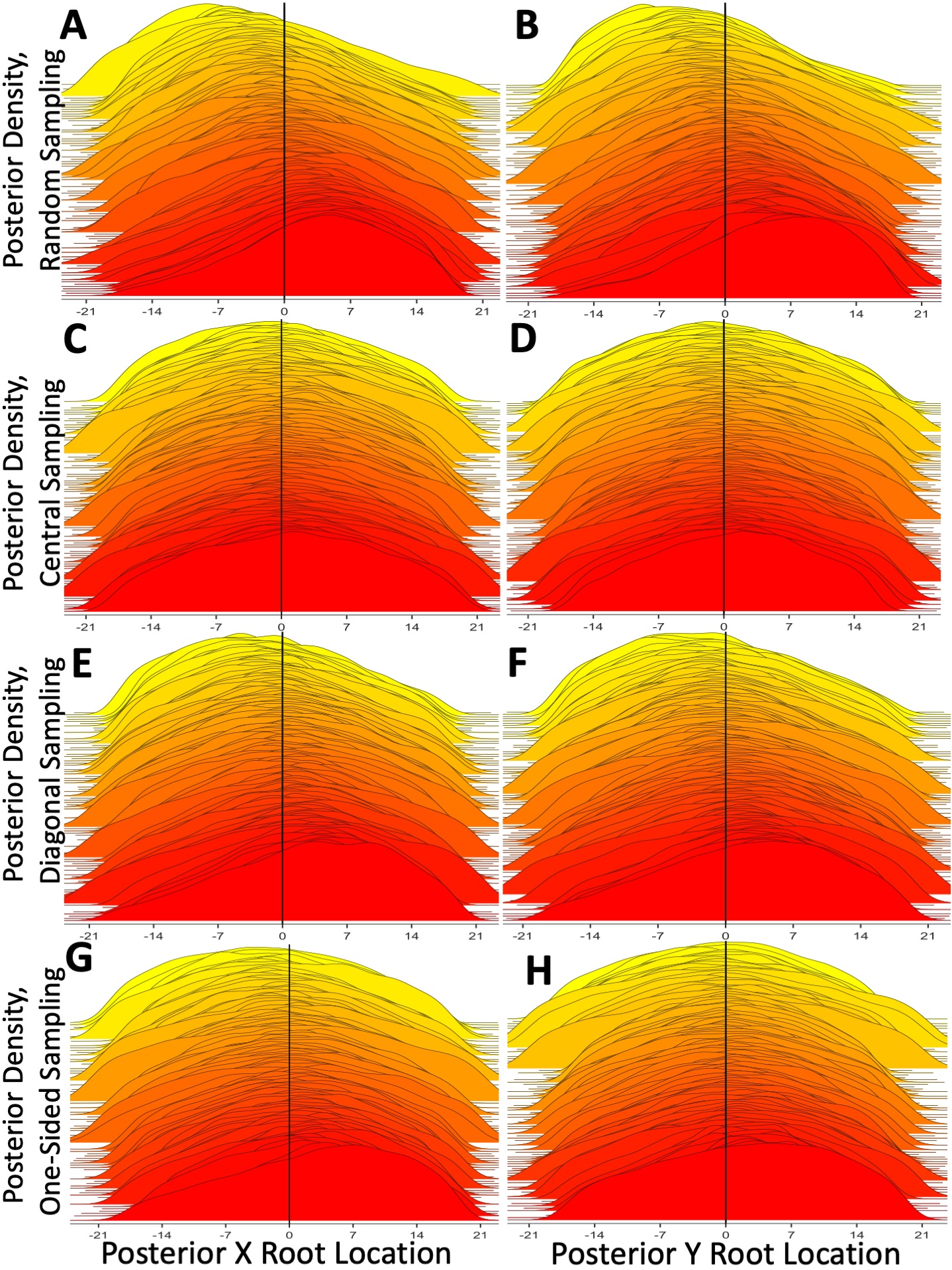

Supplement: S11 Fig — Similar to S10 Fig, but showing only converged MCMC runs (where all considered parameters have ESS>100). (JPG) [file pcbi.1008561.s012.jpg]

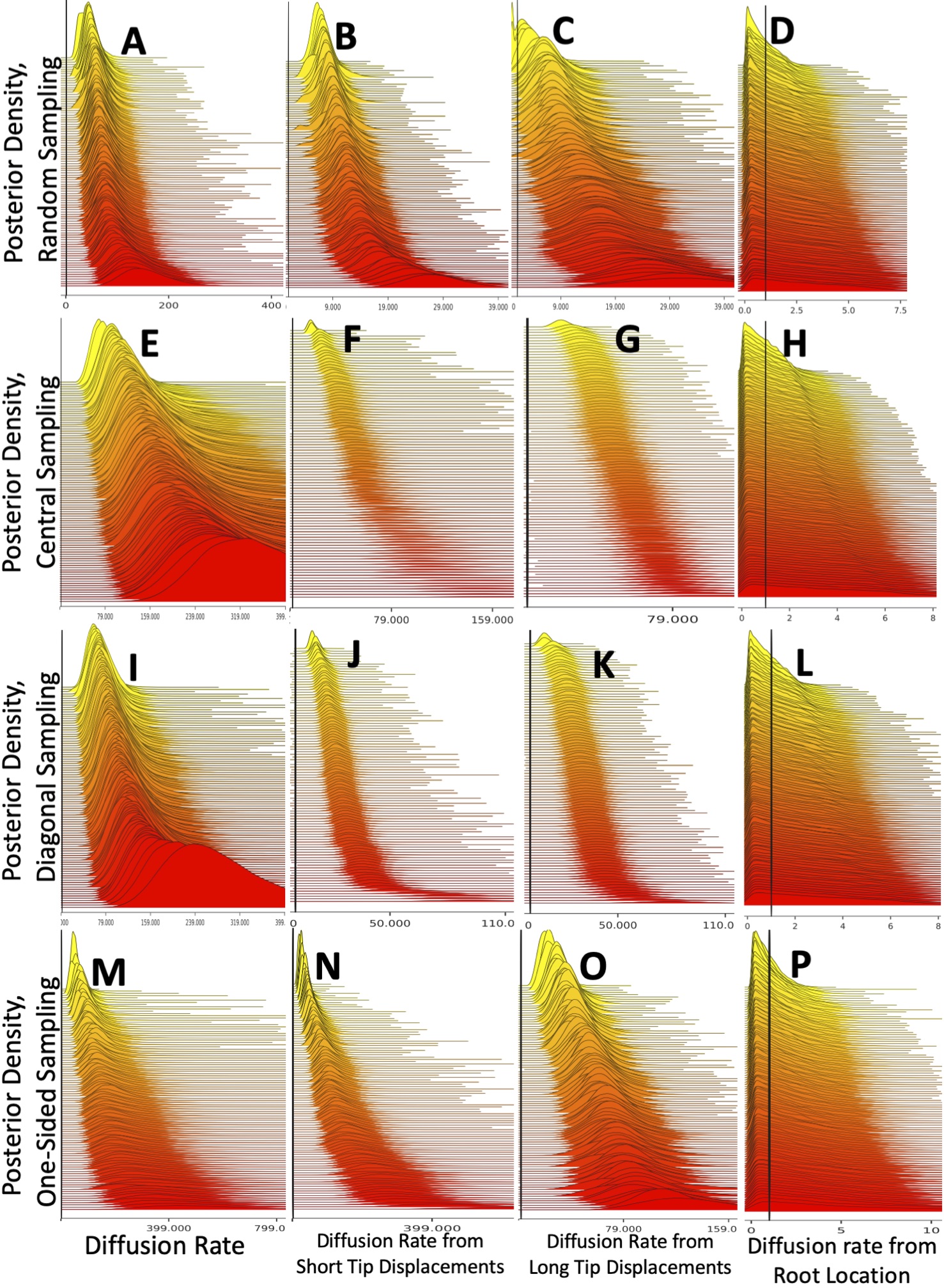

Supplement: S12 Fig — Similarly to S2 Fig, here we show inference of diffusion parameters under BMP simulations, but this time inference is performed under the ΛFV model implemented in PhyREX. Plots A,E,I,M show inferred diffusion rate σ2 using Equation 1 in S1 Text, plots B,F,J,N use method “dispersion across short distance from the tips”, plots C,G,K,O use method “dispersion across long distance from the tips”, and plots D,H,L,P use method “dispersion from the root”; see S1 Text for more details. (JPG) [file pcbi.1008561.s013.jpg]

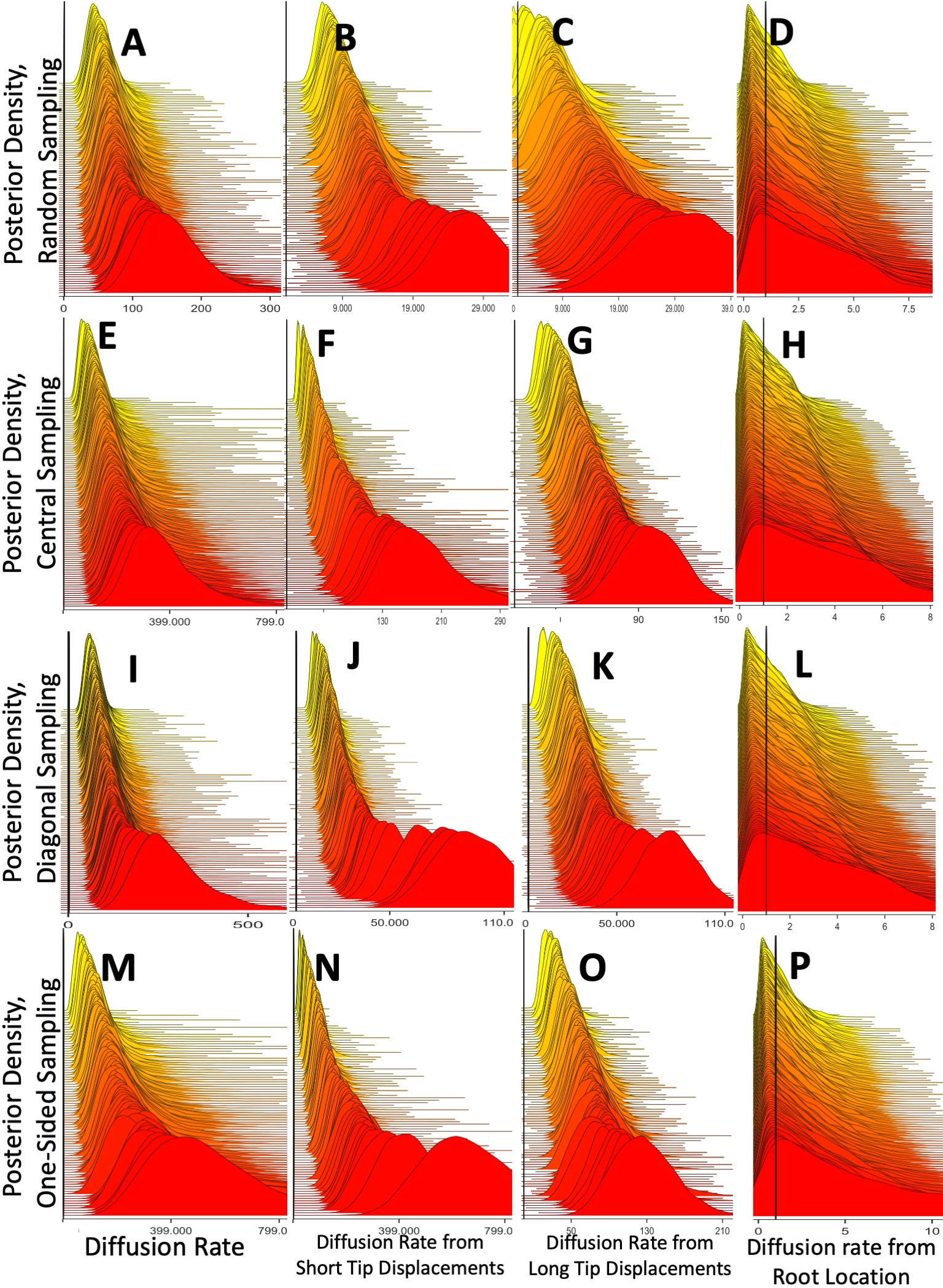

Supplement: S13 Fig — Similar to S12 Fig, but showing only converged MCMC runs (where all considered parameters have ESS>100). (JPG) [file pcbi.1008561.s014.jpg]

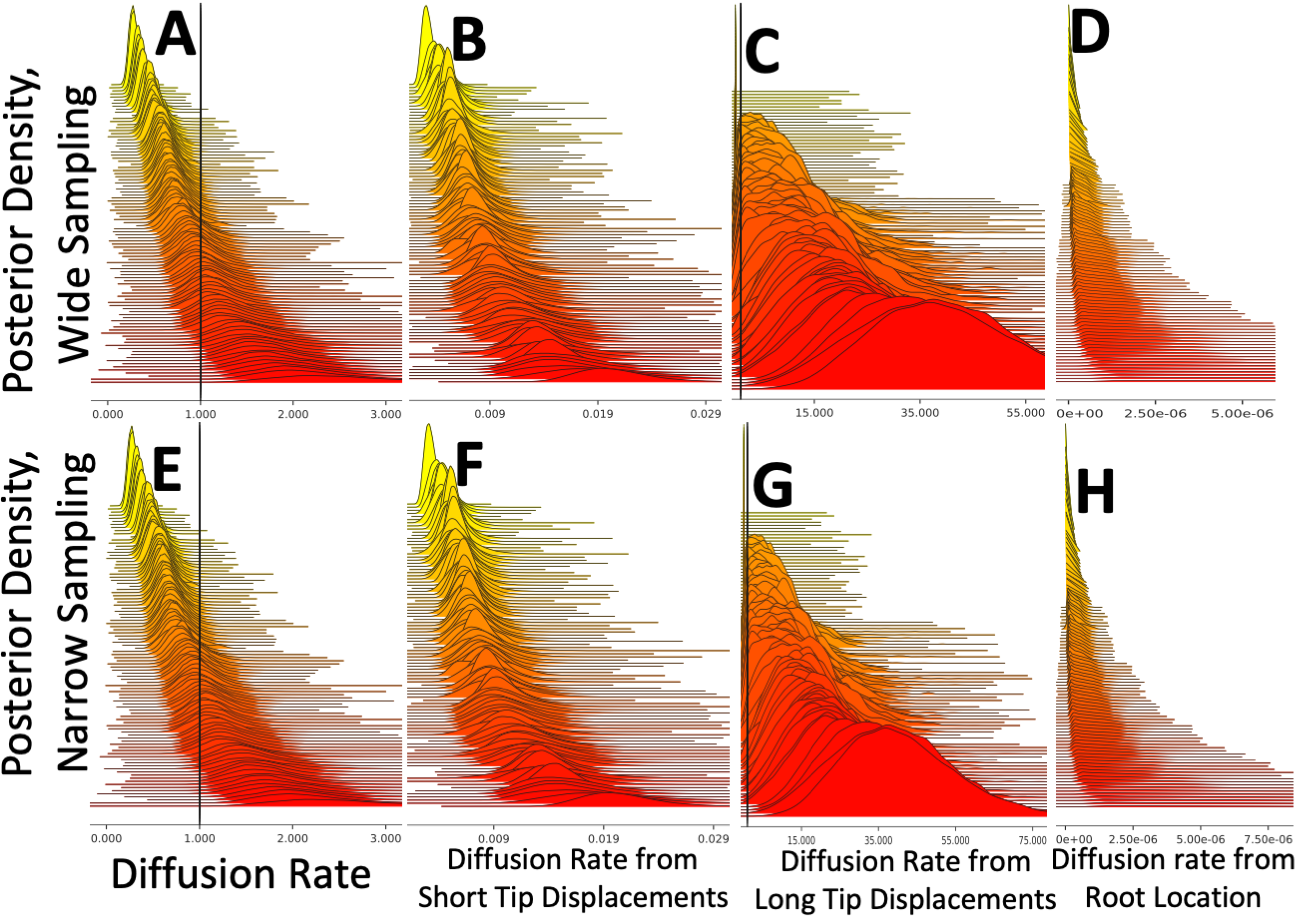

Supplement: S14 Fig — Similarly to S12 Fig, here we show PhyREX inference of diffusion parameters, but this time simulations are performed under the ΛFV model implemented in discsim. (PNG) [file pcbi.1008561.s015.png]

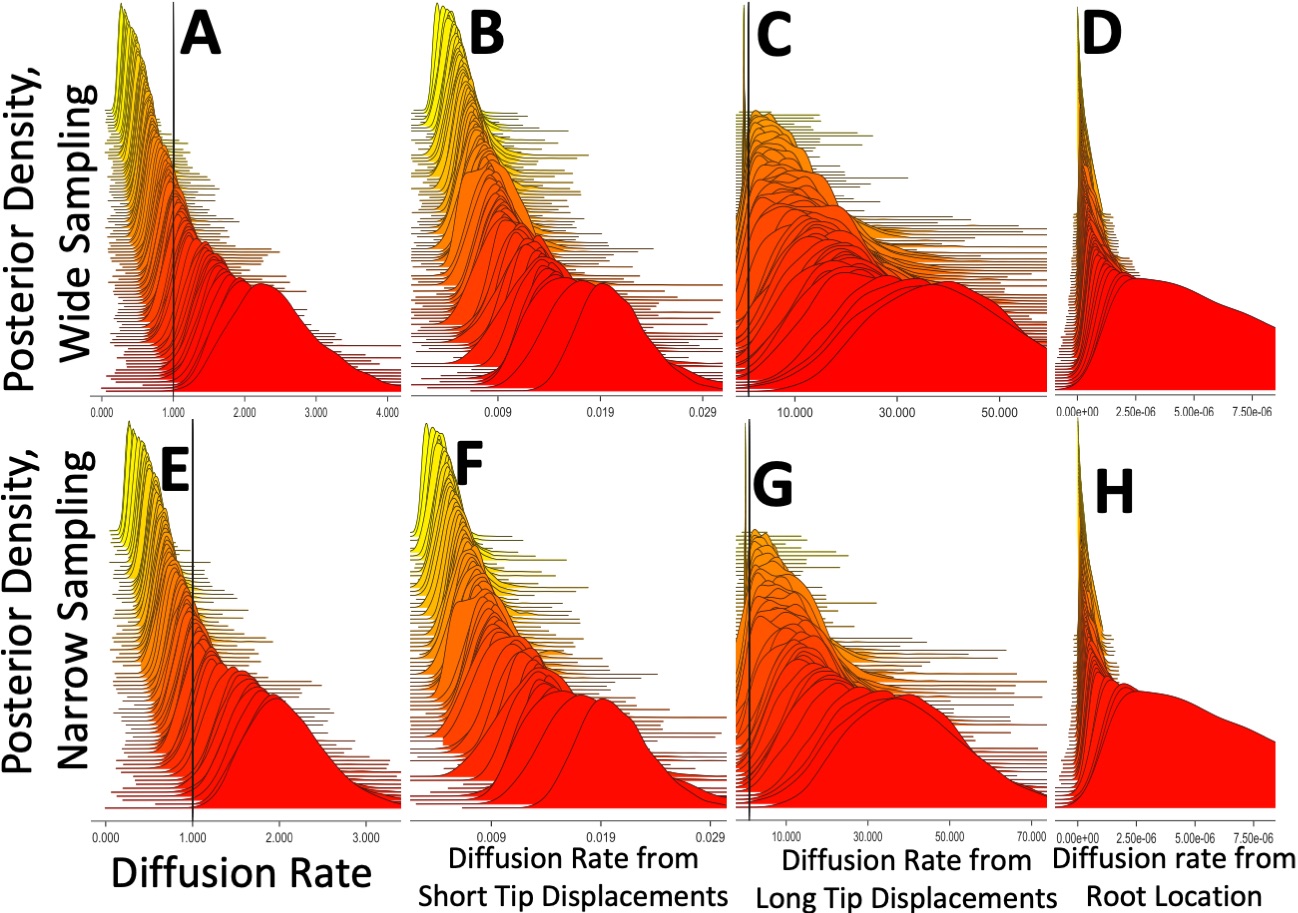

Supplement: S15 Fig — Similar to S14 Fig, but showing only converged MCMC runs (where all considered parameters have ESS>100). (JPG) [file pcbi.1008561.s016.jpg]

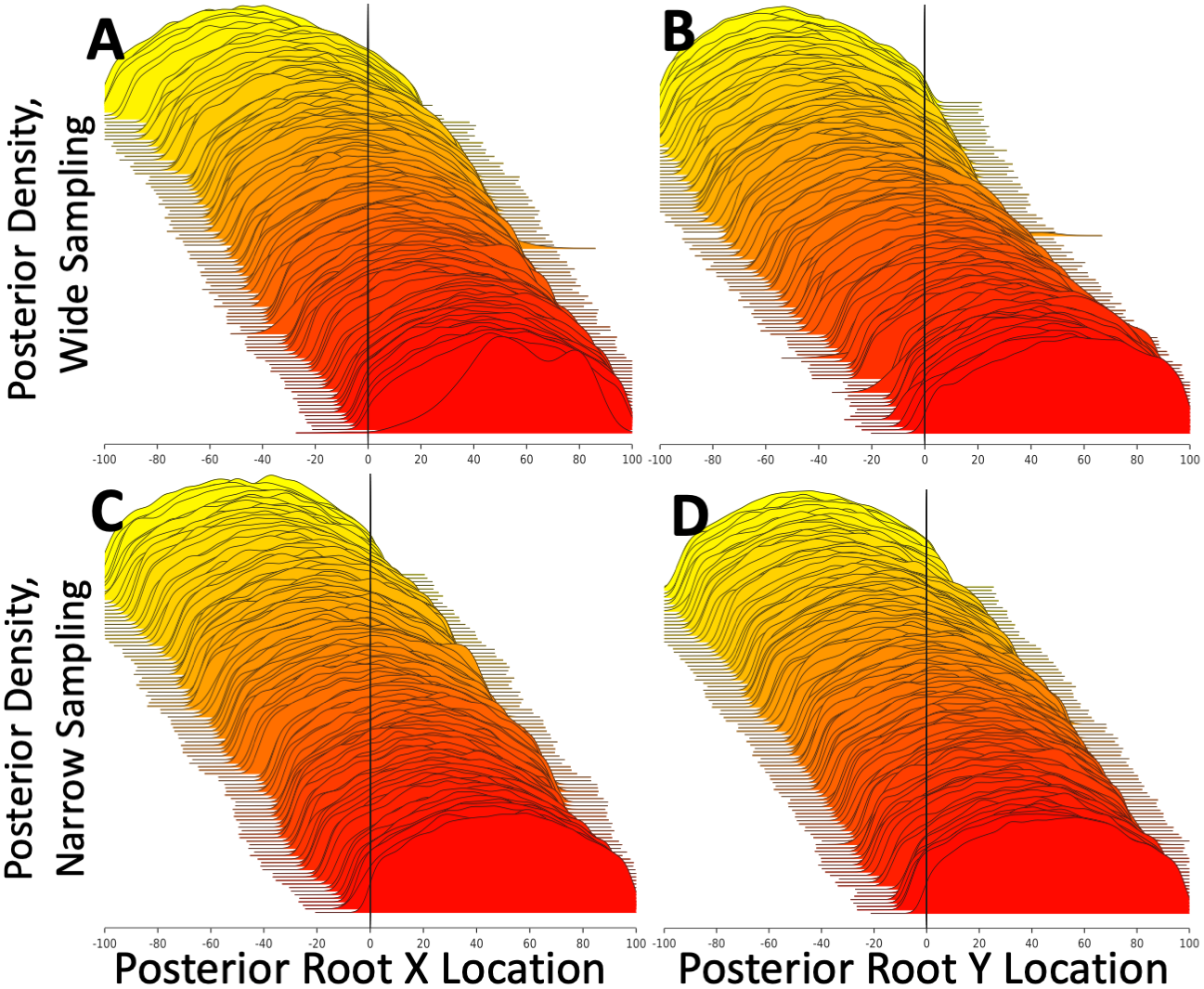

Supplement: S16 Fig — Similarly to S10 Fig, here we show PhyREX inference of root locations, but this time simulations are performed under the ΛFV model implemented in discsim. (PNG) [file pcbi.1008561.s017.png]

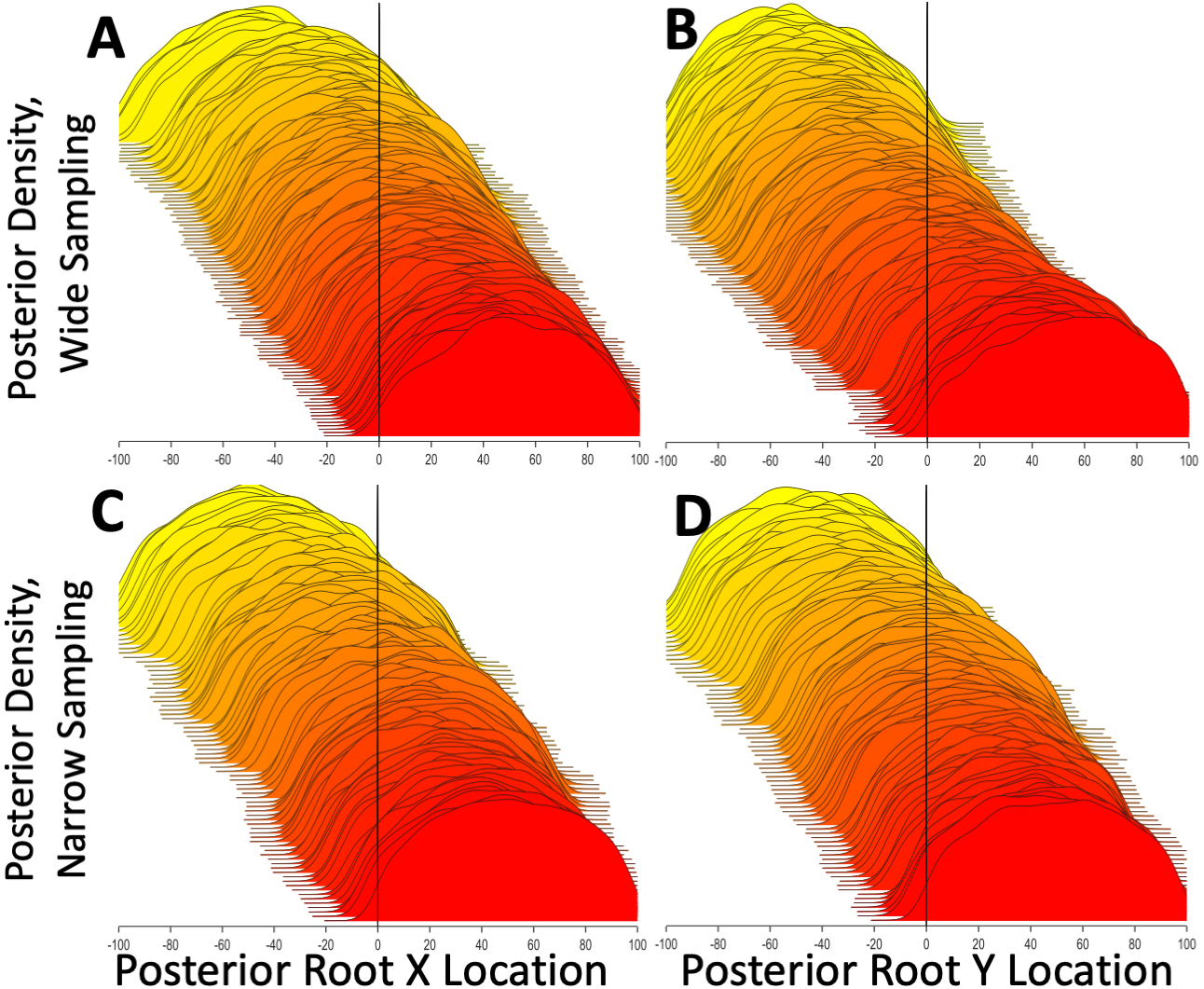

Supplement: S17 Fig — Similar to S16 Fig, but showing only converged MCMC runs (where all considered parameters have ESS>100). (PNG) [file pcbi.1008561.s018.png]

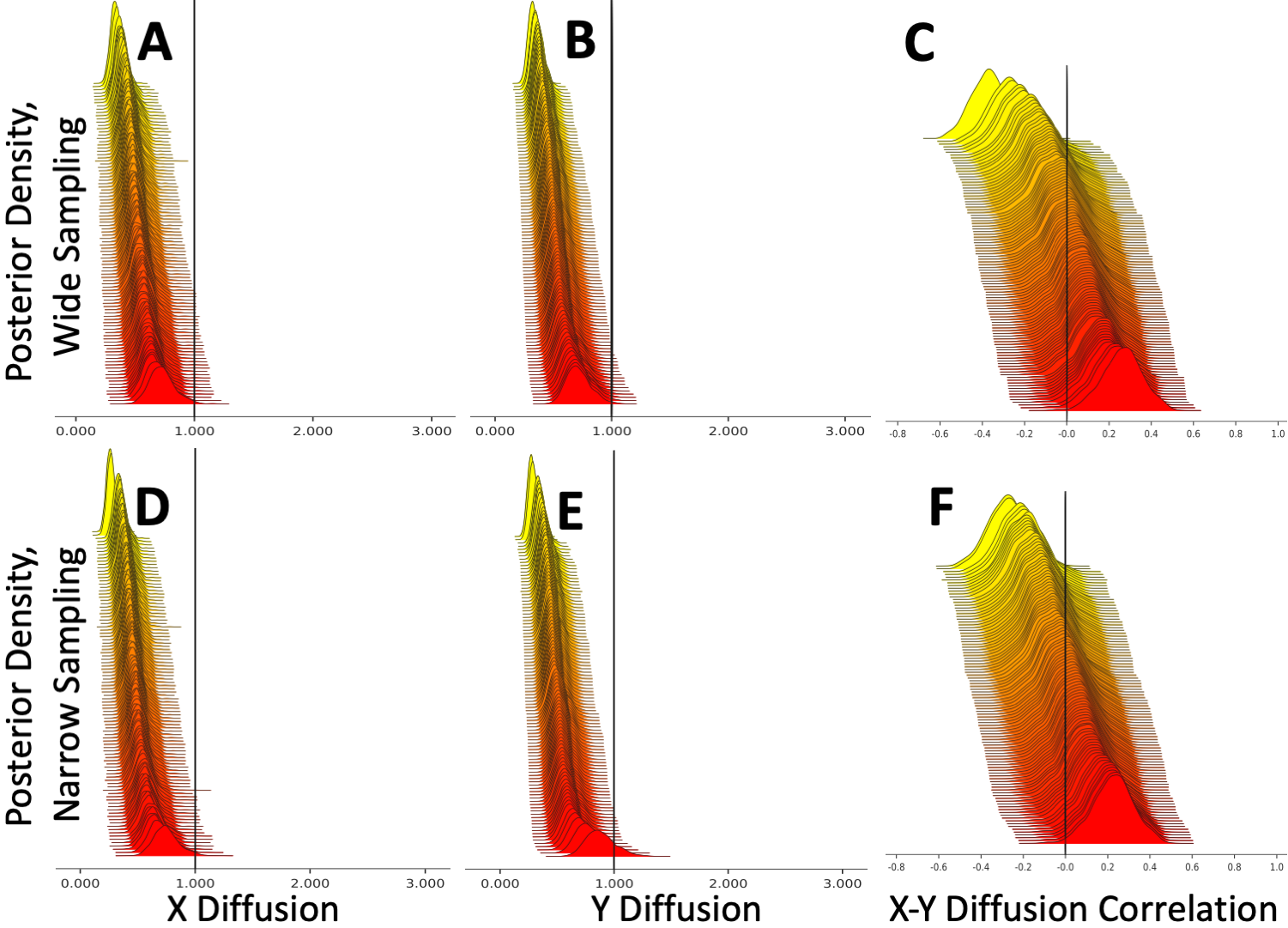

Supplement: S18 Fig — Here the BEAST BMP model was used for inference, while the discsim ΛFV model was used for inference. Plots show inferred posterior distributions for the diffusion rate in the X dimension (plots A,D), in the Y dimension (plots B,E), and for the correlation between the diffusion in the two dimensions (plots C,F). In each plot, the 100 distributions represent 100 independent replicates, and are vertically sorted based on the posterior median. Vertical black lines show the true, simulated values (in this case 1 for rates and 0 for the correlation). Plots A-C are from simulations with wide sampling, while plots D-F are with narrow sampling. (PNG) [file pcbi.1008561.s019.png]

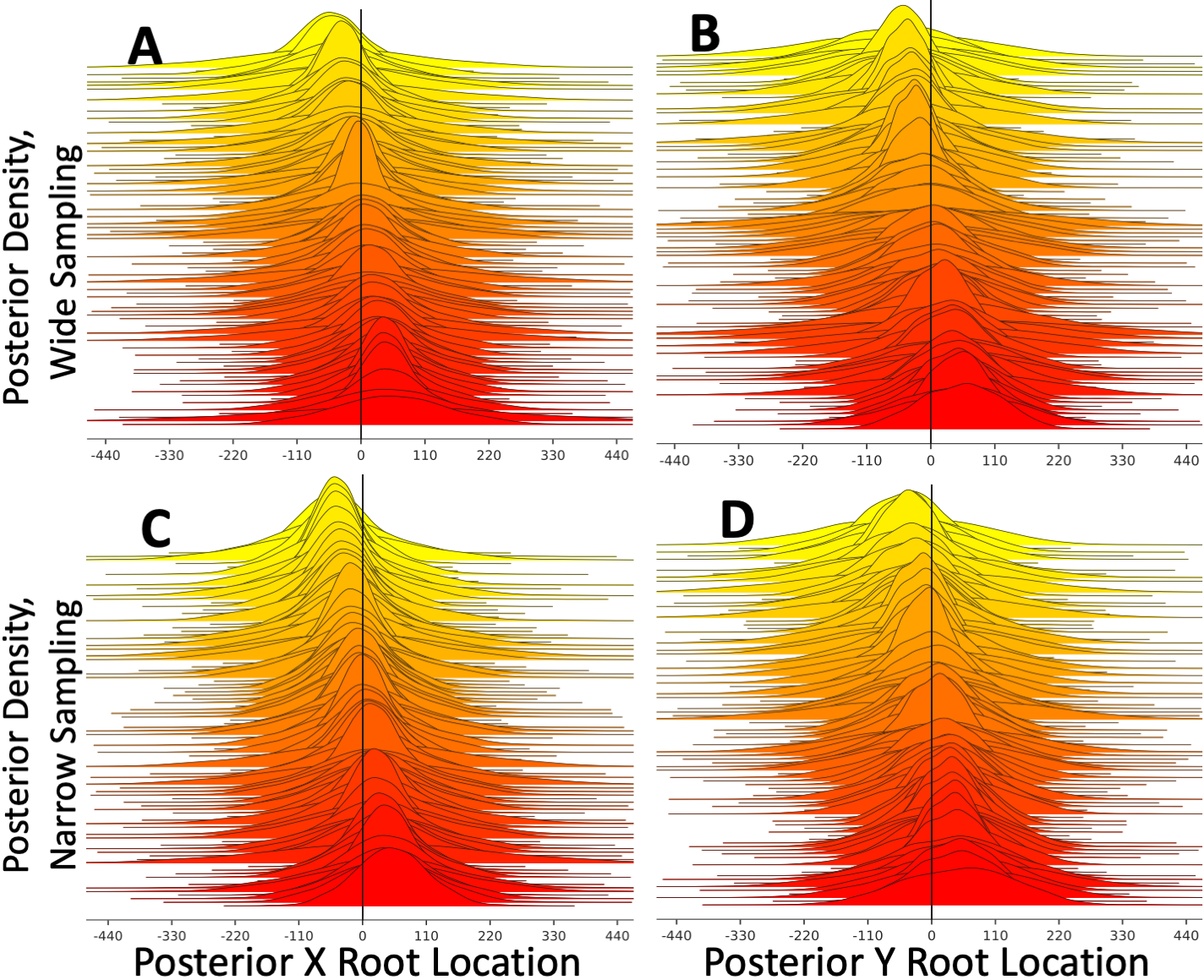

Supplement: S19 Fig — Here the BEAST BMP model was used for inference, while the discsim ΛFV model was used for inference. Plots show inferred posterior distributions for the X dimension position of the tree root (plots A,C), and its Y dimension position (plots B,D). In each plot, the 100 distributions represent 100 independent replicates, and are vertically sorted based on the posterior median. Vertical black lines show the true, simulated values (in this case always 0). Plots A,B are from simulations with wide sampling, while plots C,D are from simulations with narrow sampling. Since in many cases the MRCA of the collected samples is not the root of the whole simulated phylogeny (which was simulated at location (0, 0)), in each simulation all locations are translated (in mathematical sense) so that the true simulated sample MRCA is always at (0, 0). (PNG) [file pcbi.1008561.s020.png]

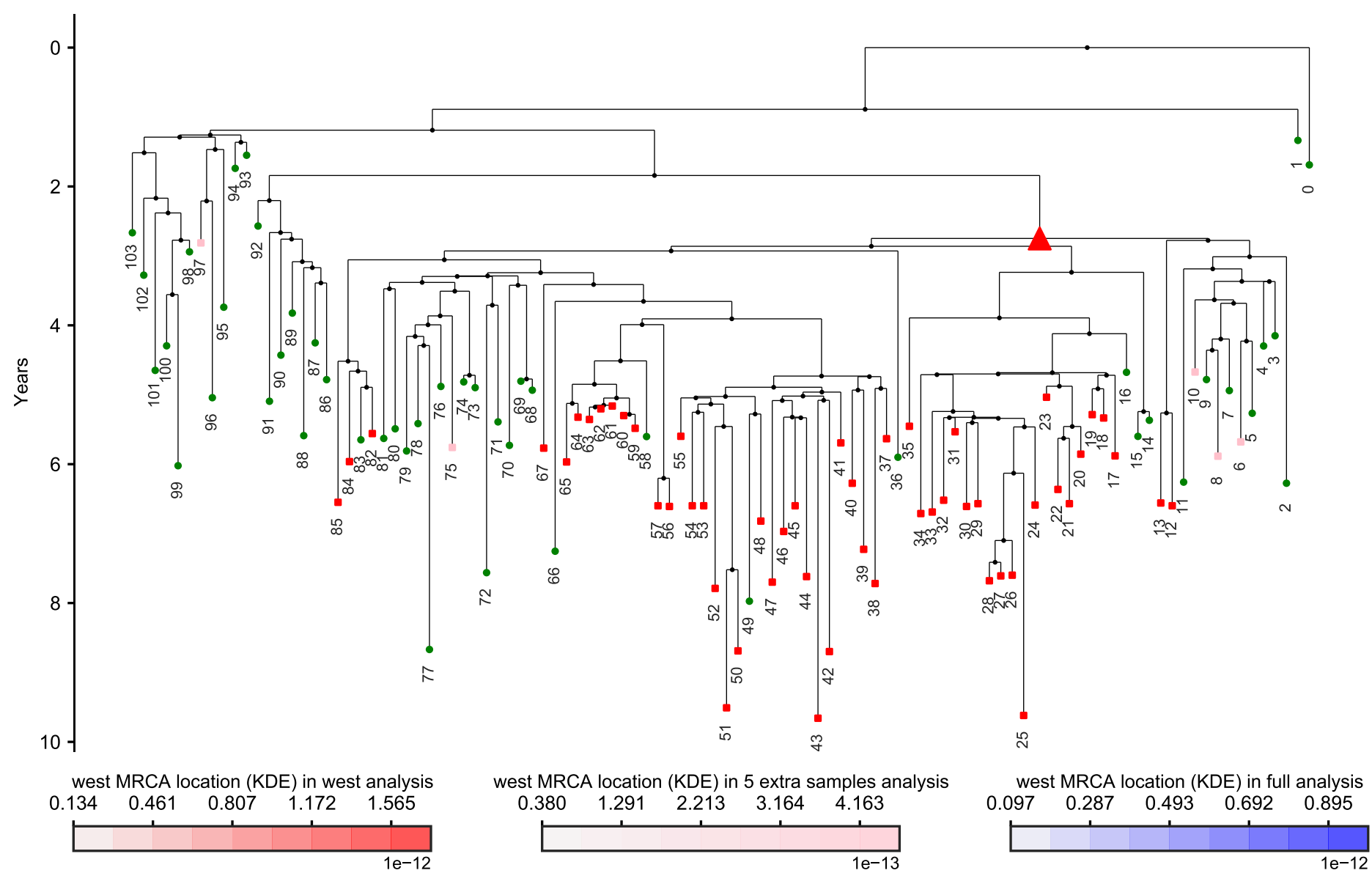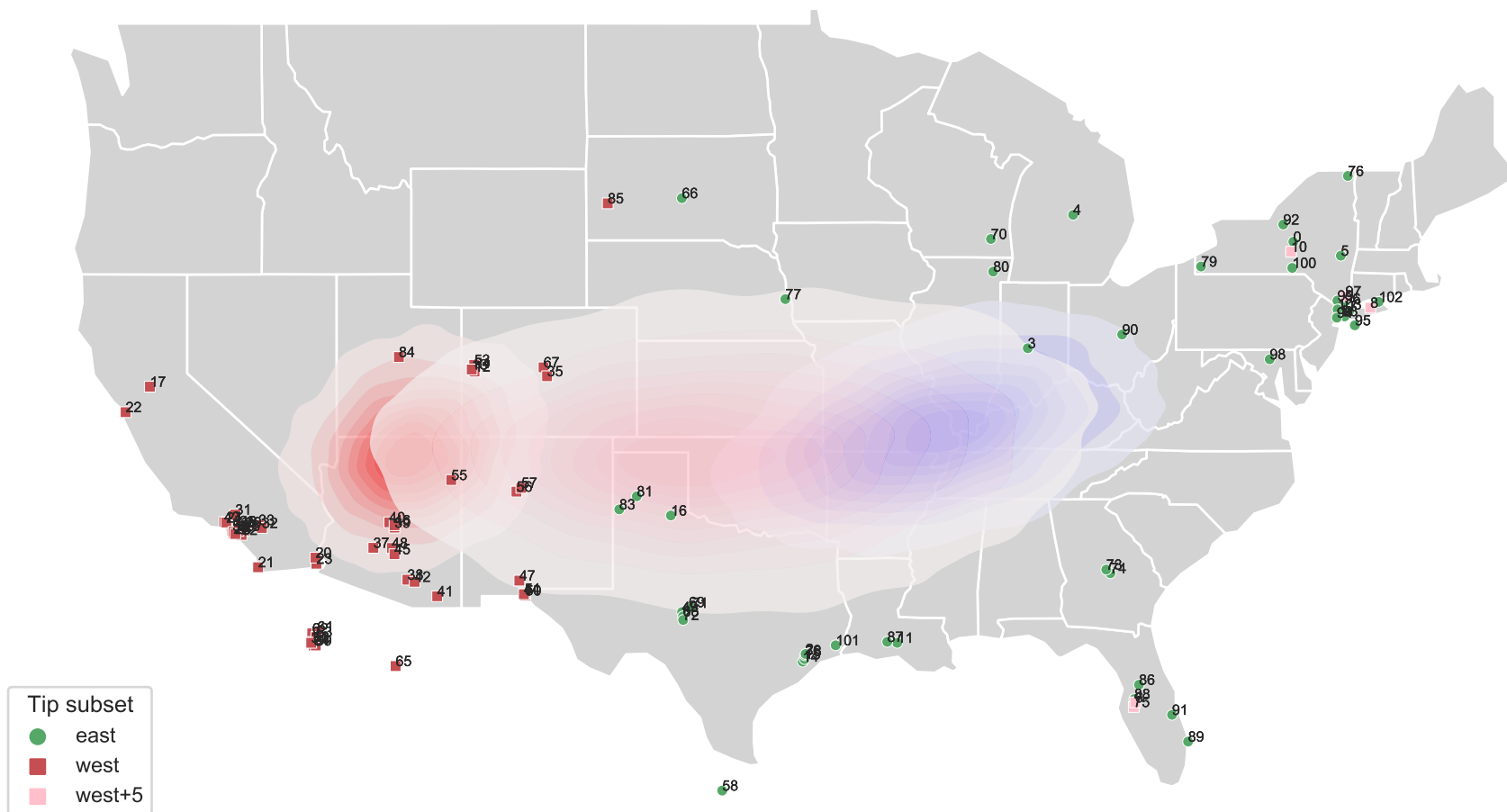

Supplement: S20 Fig — Similarly as in Fig 4, we recreate sampling bias in a West Nile Virus dataset by comparing the inference between the full dataset and the same dataset after excluding eastern samples. For all analyses we compare the inference of the ancestral location of the MRCA of all western samples. Here, we consider three analyses: the one with all samples (blue kernel density on the map), the one with only western samples (red kernel density), and one with western samples plus 5 random eastern samples (pink kernel density) representing a scenario of strong, but not extreme, sampling bias. The 5 random eastern samples are also represented in the phylogeny as pink tips. (PDF) [file pcbi.1008561.s021.pdf]

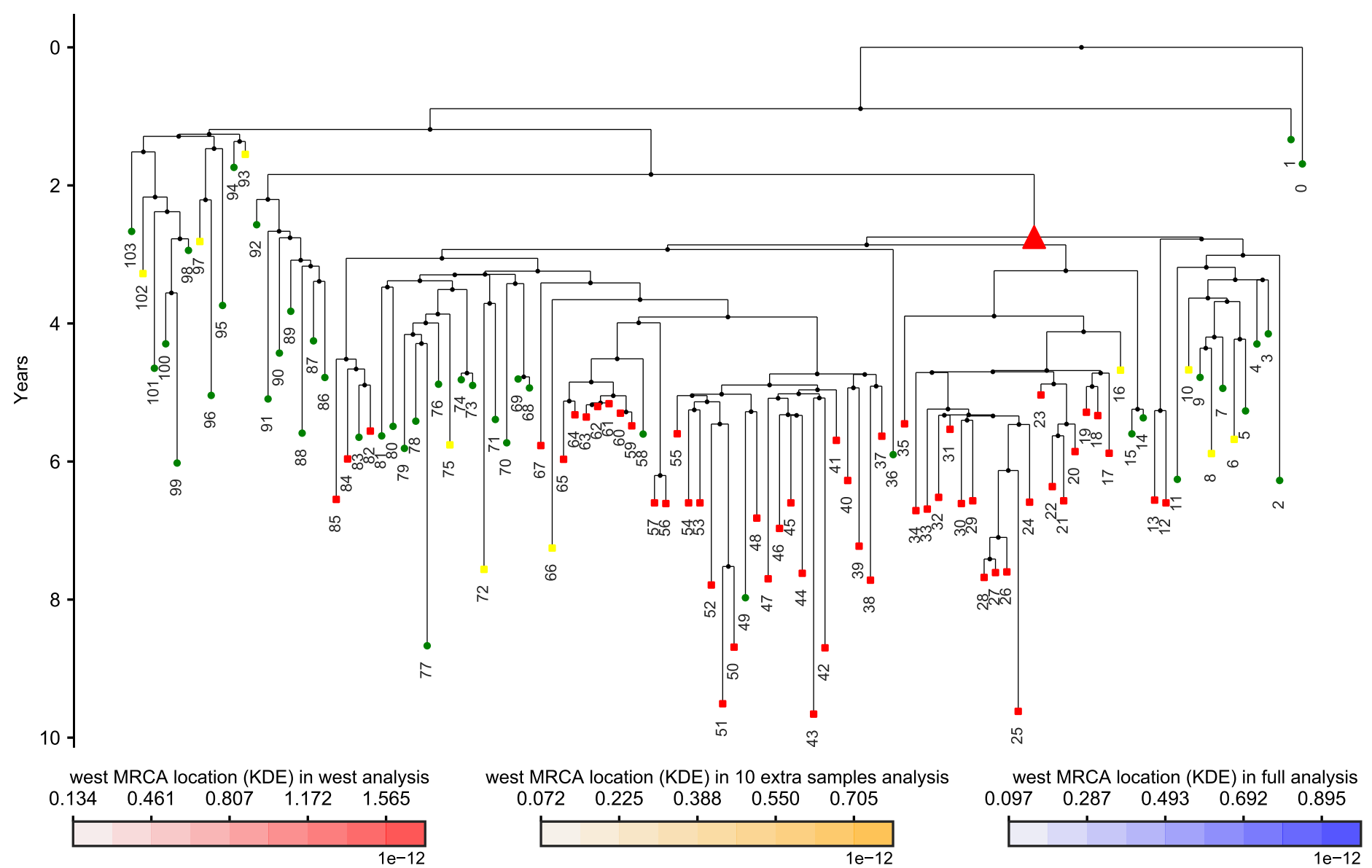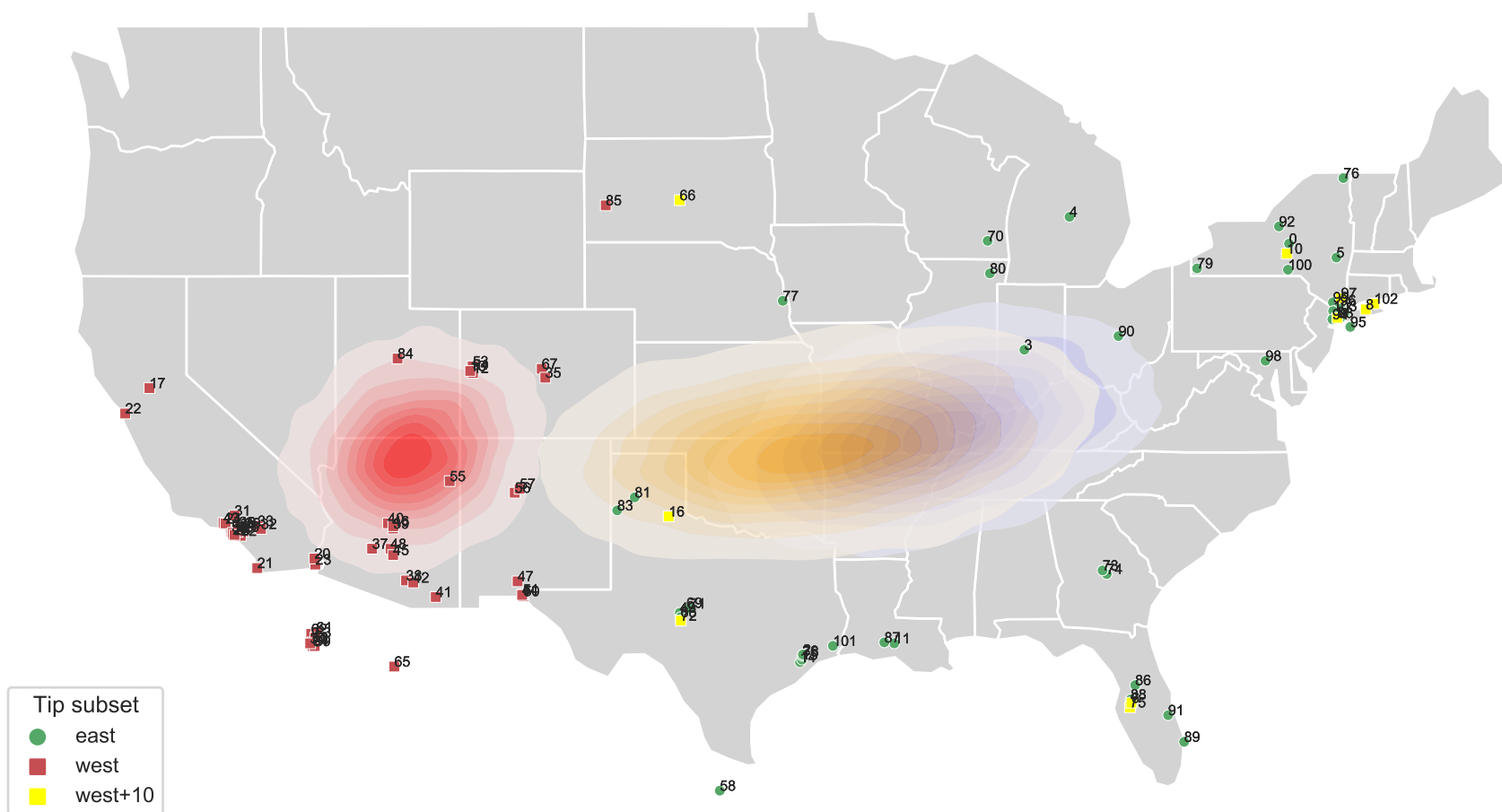

Supplement: S21 Fig — Same as S20 Fig, but this time with 10 extra eastern samples instead of 10 (yellow tips and orange kernel density), representing a scenario of moderate sampling bias. (PDF) [file pcbi.1008561.s022.pdf]

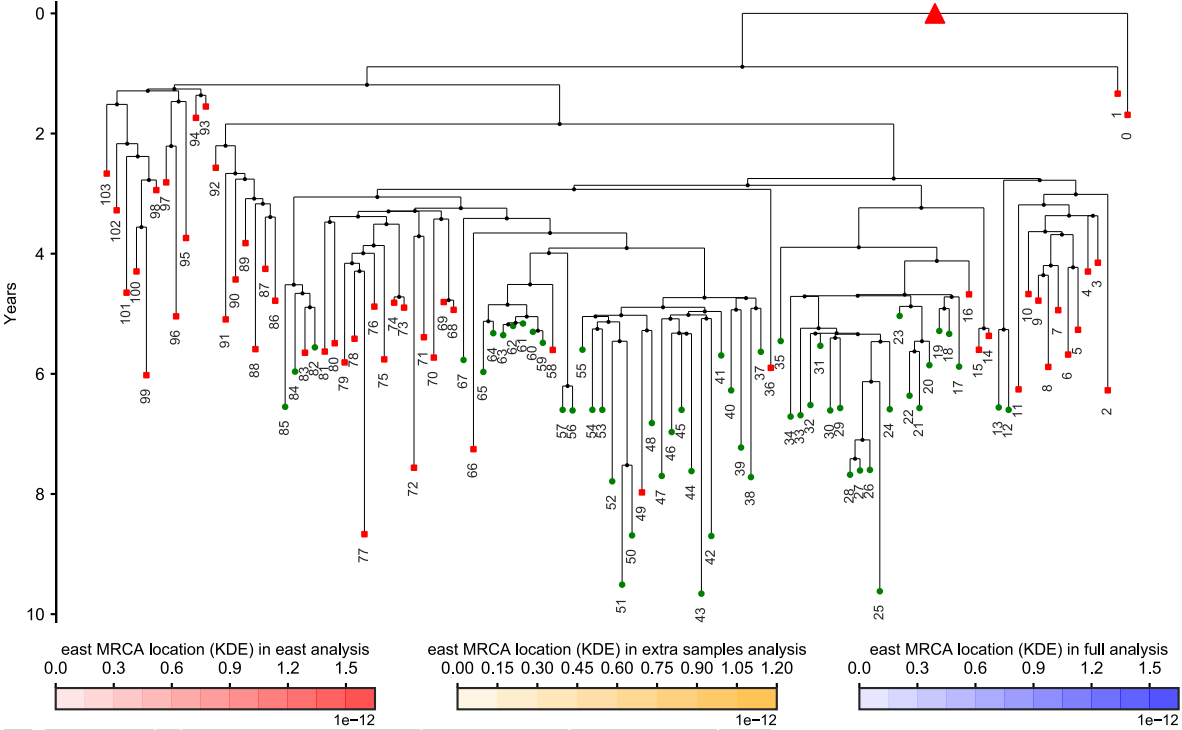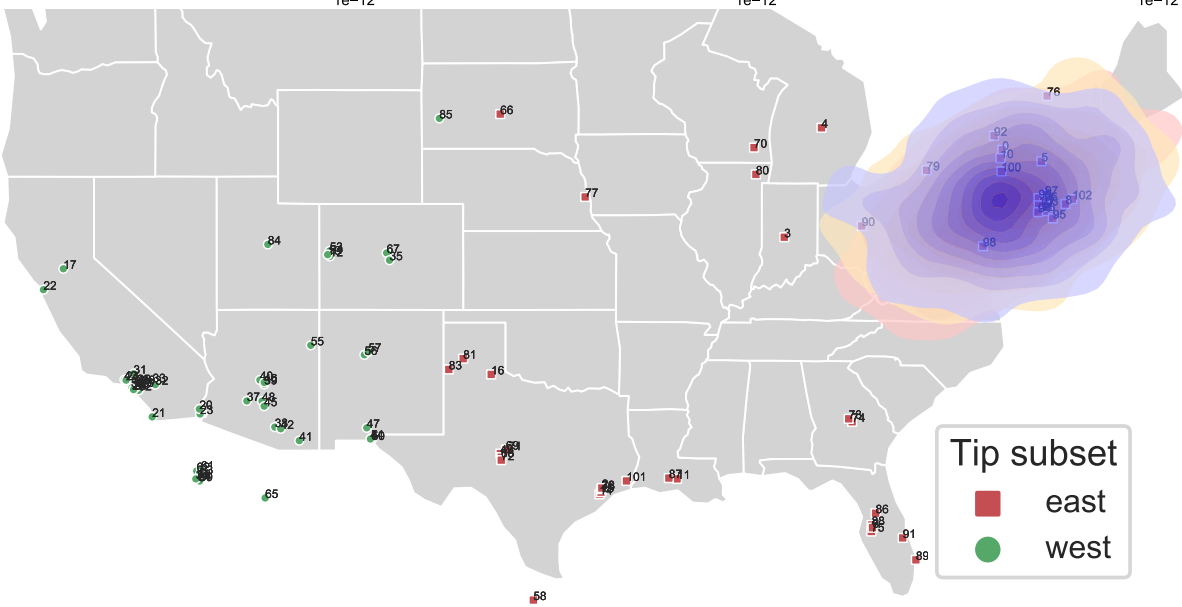

Supplement: S22 Fig — We re-analysed the West Nile Virus North America dataset of Pybus and colleagues [14] as in the main text, but this time selecting only the east-most samples. At top, we show the maximum clade credibility tree from the full dataset. Branch lengths are in years. Green circles represent western samples while red squares represent eastern ones. The red triangle in the tree represents the node whose location is considered here: the most recent common ancestor (MRCA) of all samples. Below, the sample locations are shown on a map of the USA. Sample numbers are only used to link samples on the map to samples on the phylogeny. All three kernel density estimate areas (red, orange and blue) on the map represent the posterior densities of the location of the MRCA (red triangle in the phylogeny). The red area represents the posterior from the analysis of only eastern samples; the blue area is the posterior from the analysis of all samples; the orange area is the posterior from the analysis of the eastern samples and of sequence-free western samples (western samples included but without sequence data). (PDF) [file pcbi.1008561.s023.pdf]

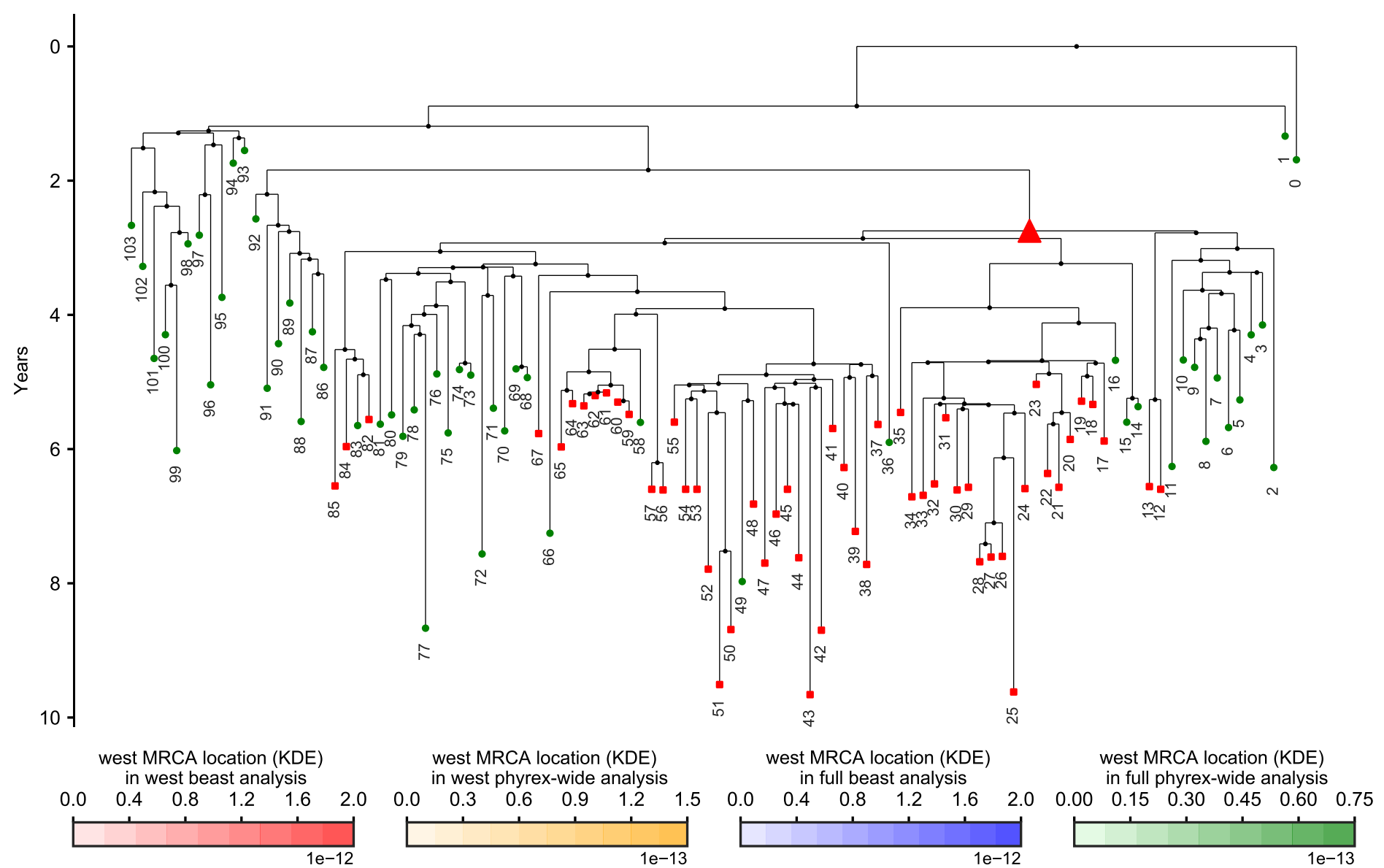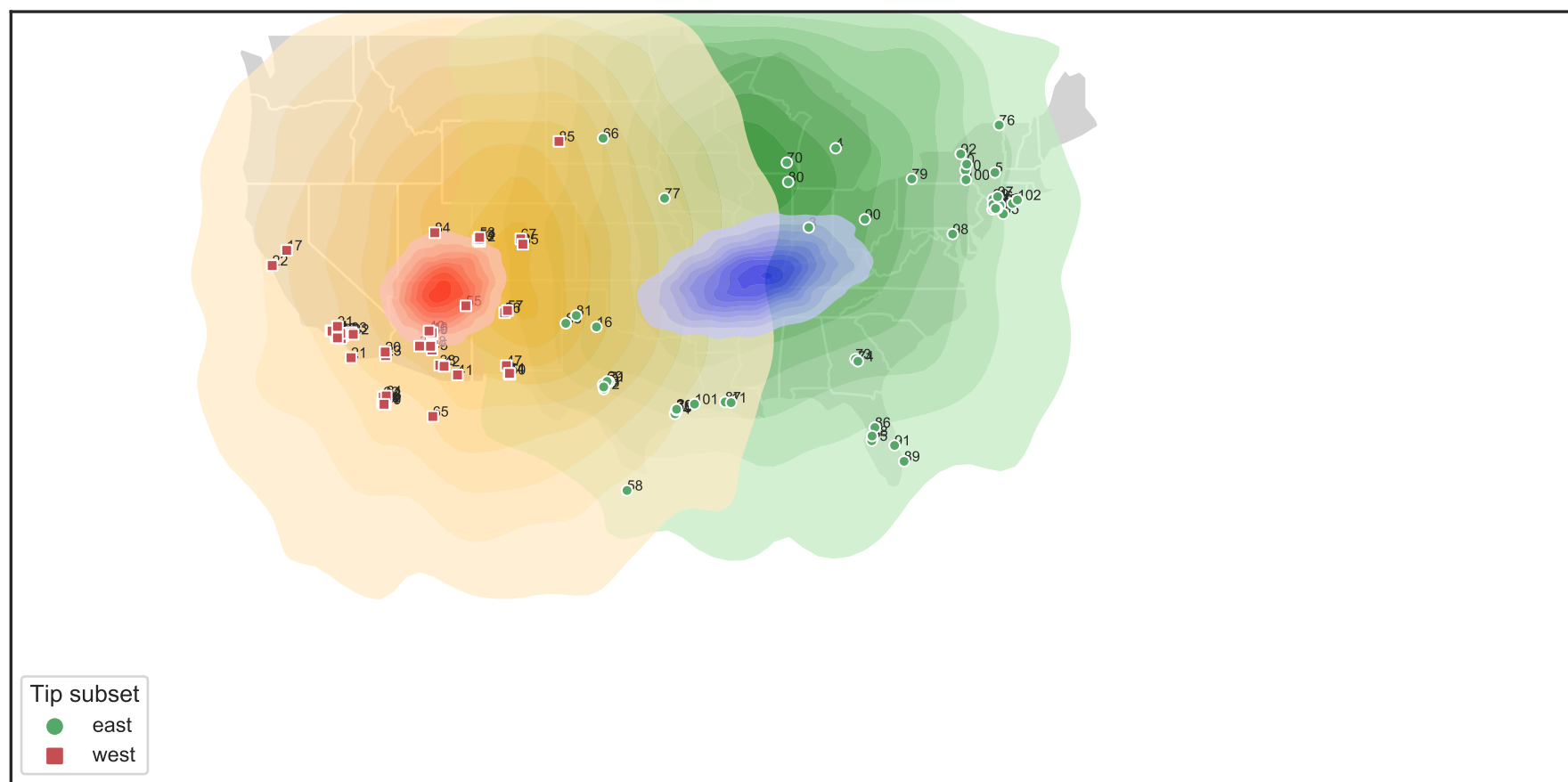

Supplement: S23 Fig — Same analysis as in Fig 5, but using a broader rectangular space (outlined in black) in PhyREX, latitude interval [6, 50] and longitude interval [−140, −35]. Posterior distribution of the diffusion rate inferred by PhyREX has a mean of 389.2 (95% HPD interval [132.4, 642.3]) km/year in the full dataset and of 150.5 ([16.6, 287.8]) km/year with only western samples. (PDF) [file pcbi.1008561.s024.pdf]

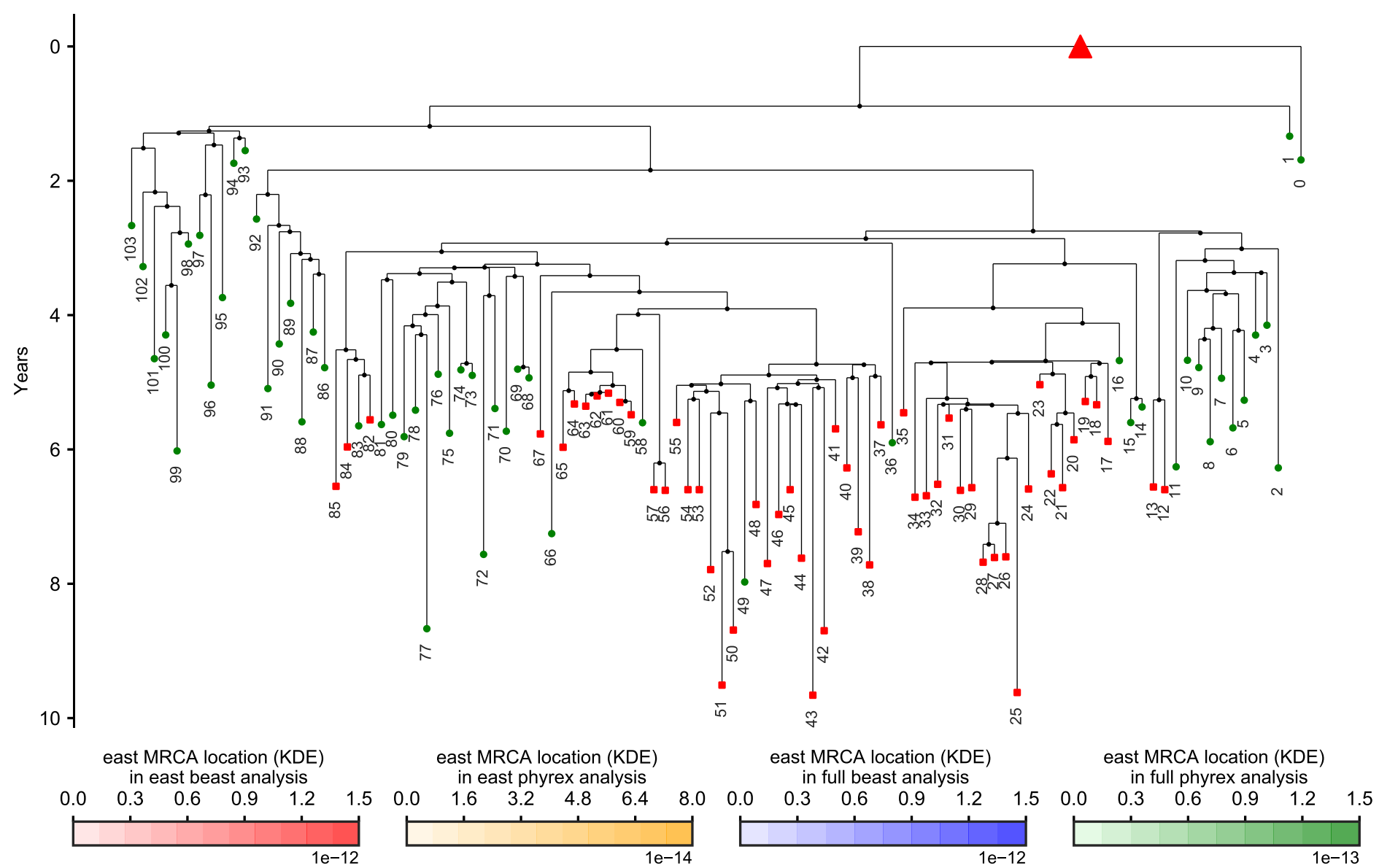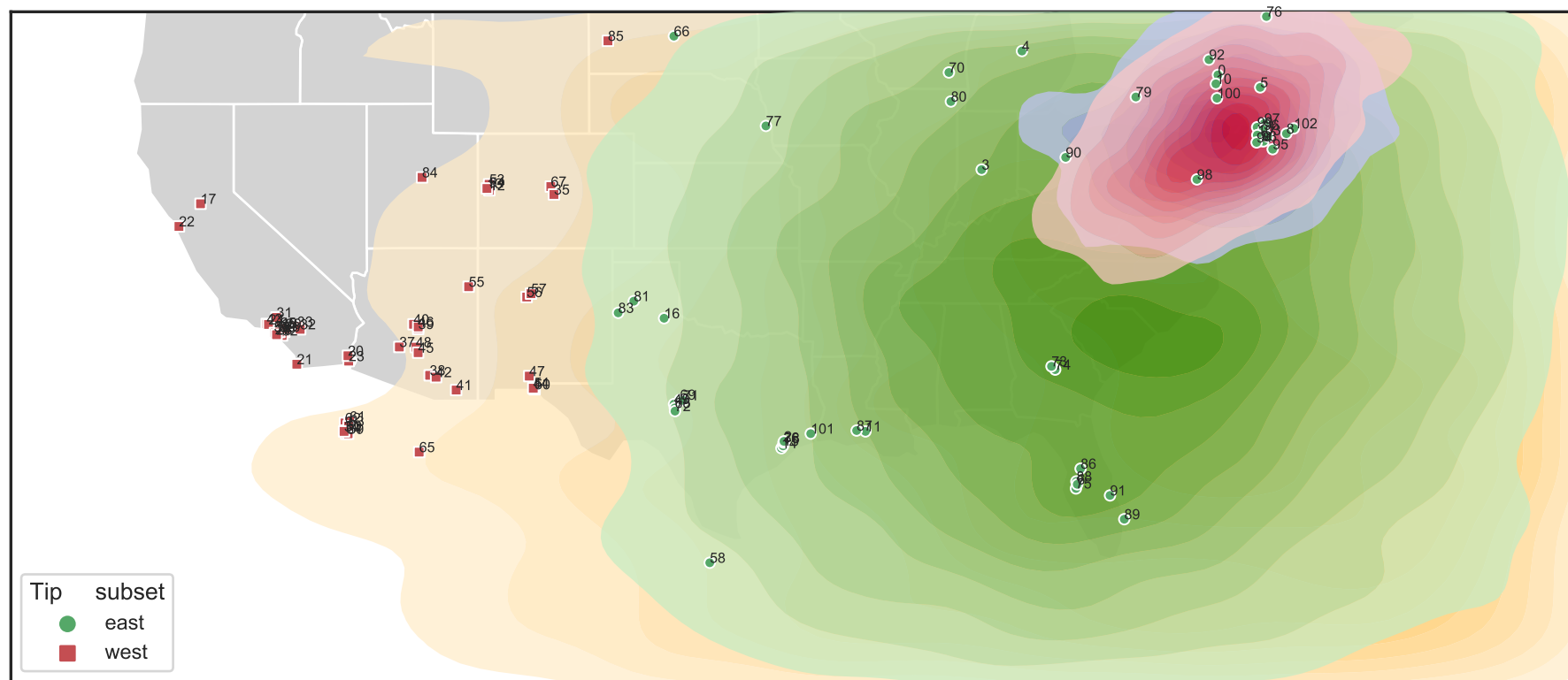

Supplement: S24 Fig — We re-analysed the dataset of S22 Fig, using PhyREX for both the full dataset and the one containing only eastern samples. In PhyREX we defined a rectangular space (outlined in black) with latitude interval [20, 45] and longitude interval [−130, −60]. The red area represents the posterior ancestral location from the analysis of only eastern samples in BEAST. the orange area is the same for PhyREX. The blue area is the posterior from the analysis of all samples in BEAST. The green area is the posterior from the analysis of all samples in PhyREX. Posterior distribution of the diffusion rate inferred by PhyREX has a mean of 329.9 (95% HPD interval [149.5, 497.4]) km/year in the full dataset and of 219.4 ([39.3, 391.5]) km/year with only eastern samples. (PDF) [file pcbi.1008561.s025.pdf]

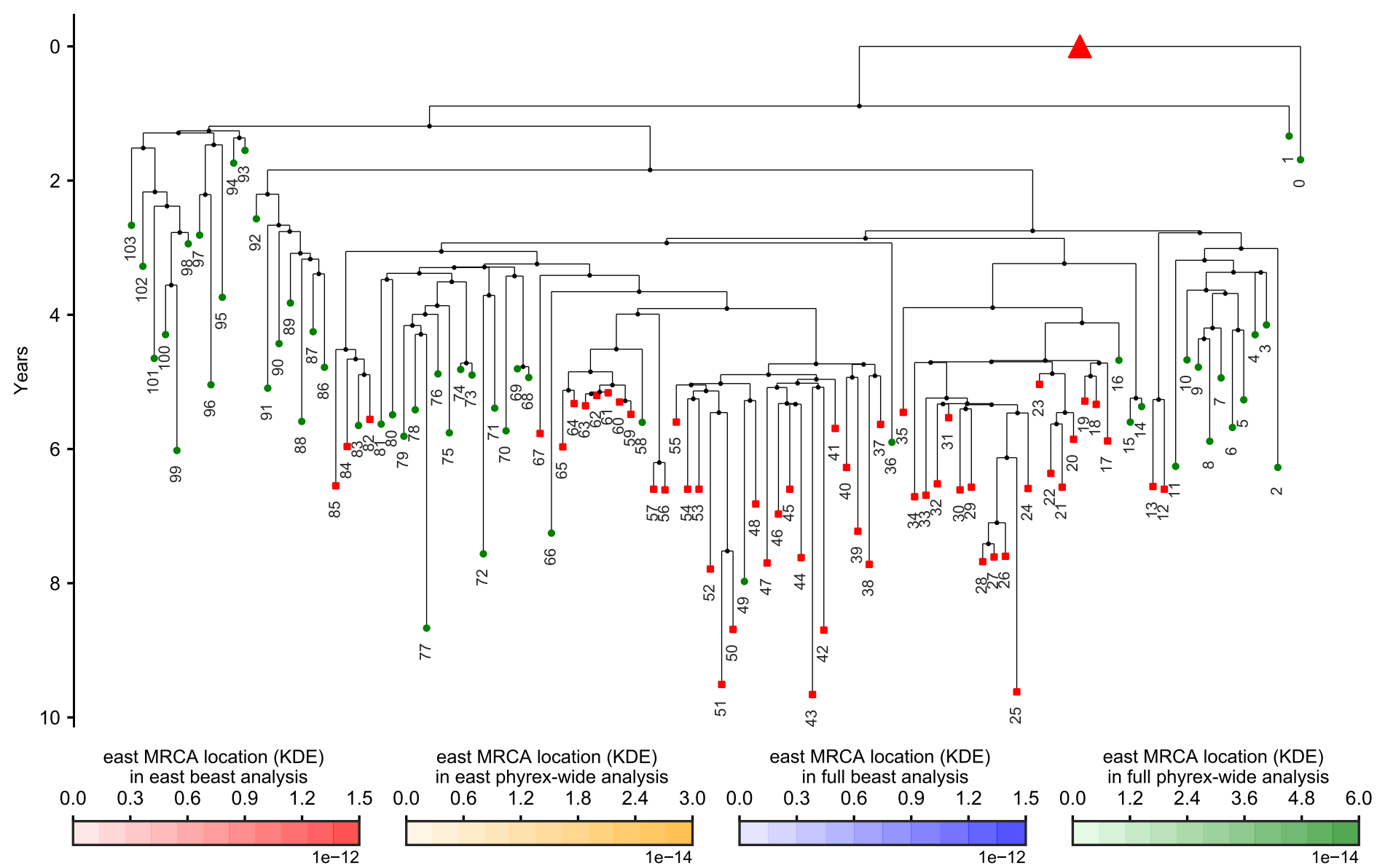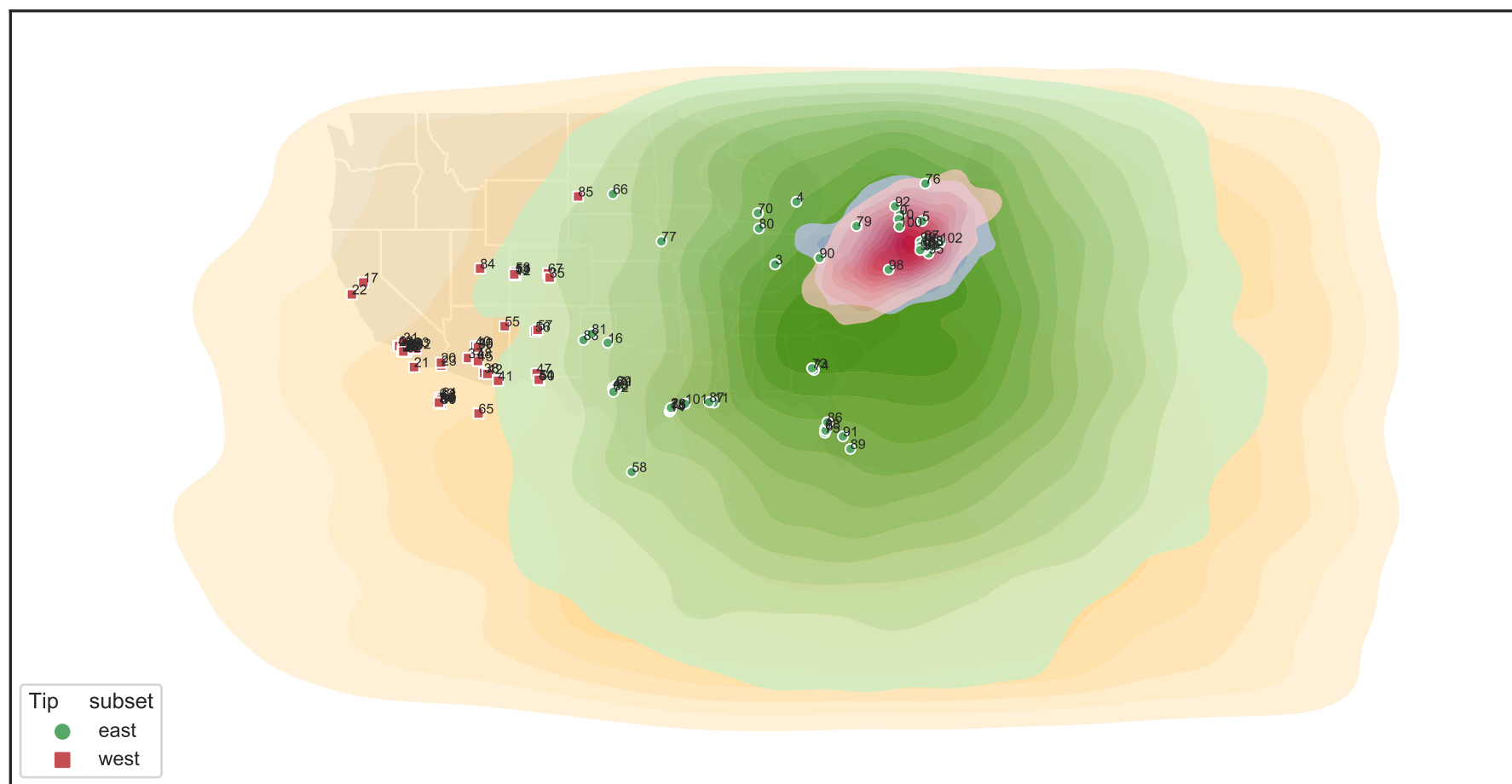

Supplement: S25 Fig — Same analysis as in S24 Fig, but using a broader rectangular space (outlined in black) in PhyREX, latitude interval [6, 50] and longitude interval [−140, −35]. Posterior distribution of the diffusion rate inferred by PhyREX has a mean of 389.2 (95% HPD interval [132.4, 642.3]) km/year in the full dataset and of 304.7 ([36.2, 580.4]) km/year with only eastern samples. (PDF) [file pcbi.1008561.s026.pdf]

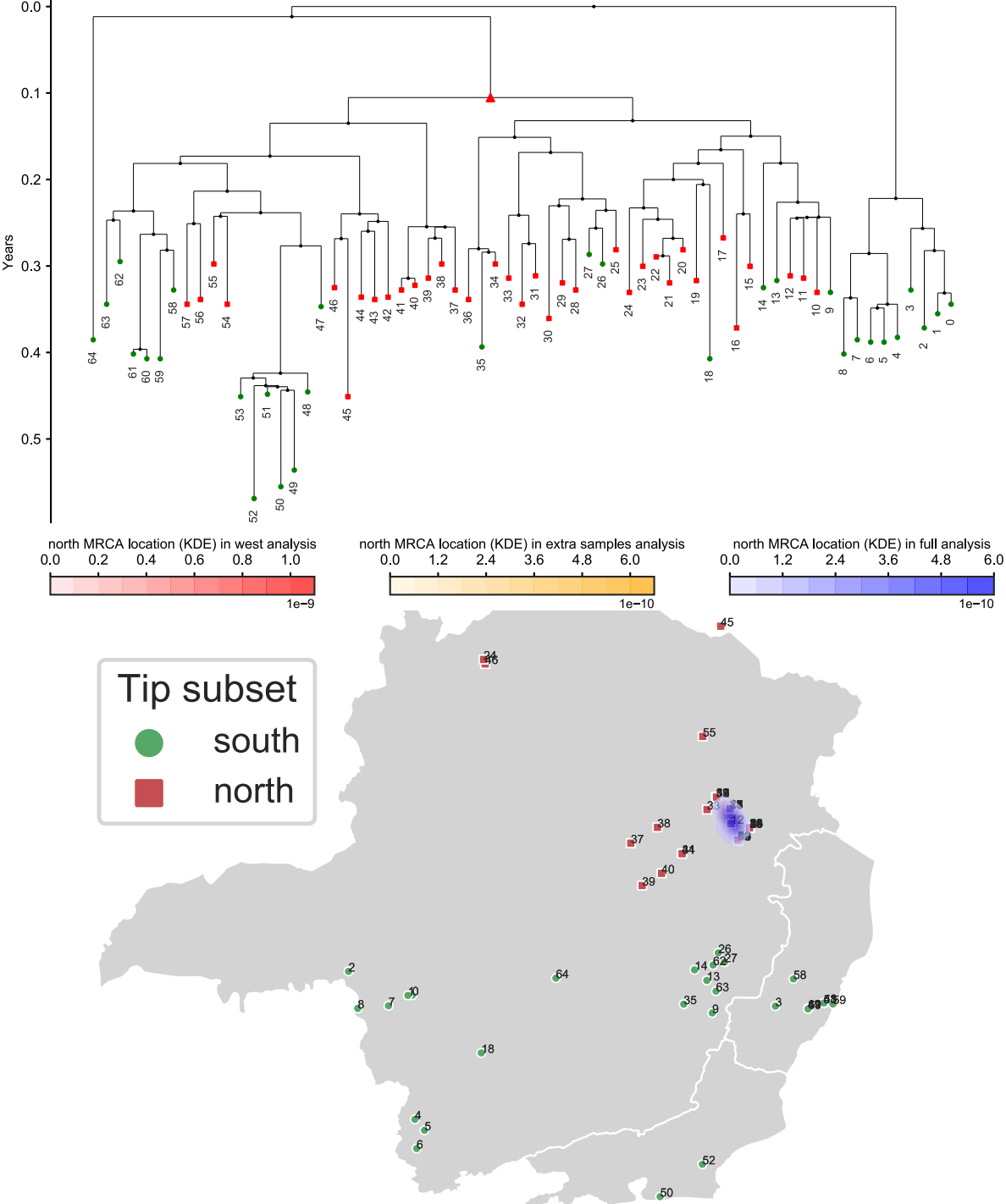

Supplement: S26 Fig — To investigate the possible effects of sampling bias, we again consider the effects or restricting an analysis to a geographical subsample of an original dataset. Here we compare the results of BMP analysis using all the data from [46] versus using only the northern samples (latitude above −19.0, red squares in the phylogeny and on the map). On top is the maximum clade credibility phylogeny inferred from analysing the whole dataset. On the map (bottom) we show the location of the samples and the inferred location of the most recent common ancestor of all southern samples (red triangle in the phylogeny). The three, almost completely overlapping colored areas on the map show the inferred posterior distribution (kernel density estimate) of the location of this ancestor from three analyses: using only the northern samples (red area), using all samples (blue area) or using only the northern samples but adding the southern ones as sequence-free samples (orange areas). The three small areas completely overlap, masking each other in the figure. A noticeable difference between the analyses is that when restricting to just the northern samples diffusion was inferred to be slower (95% HPD interval [152, 1018] km/yr versus [471, 1512] of the full analysis). (PDF) [file pcbi.1008561.s027.pdf]

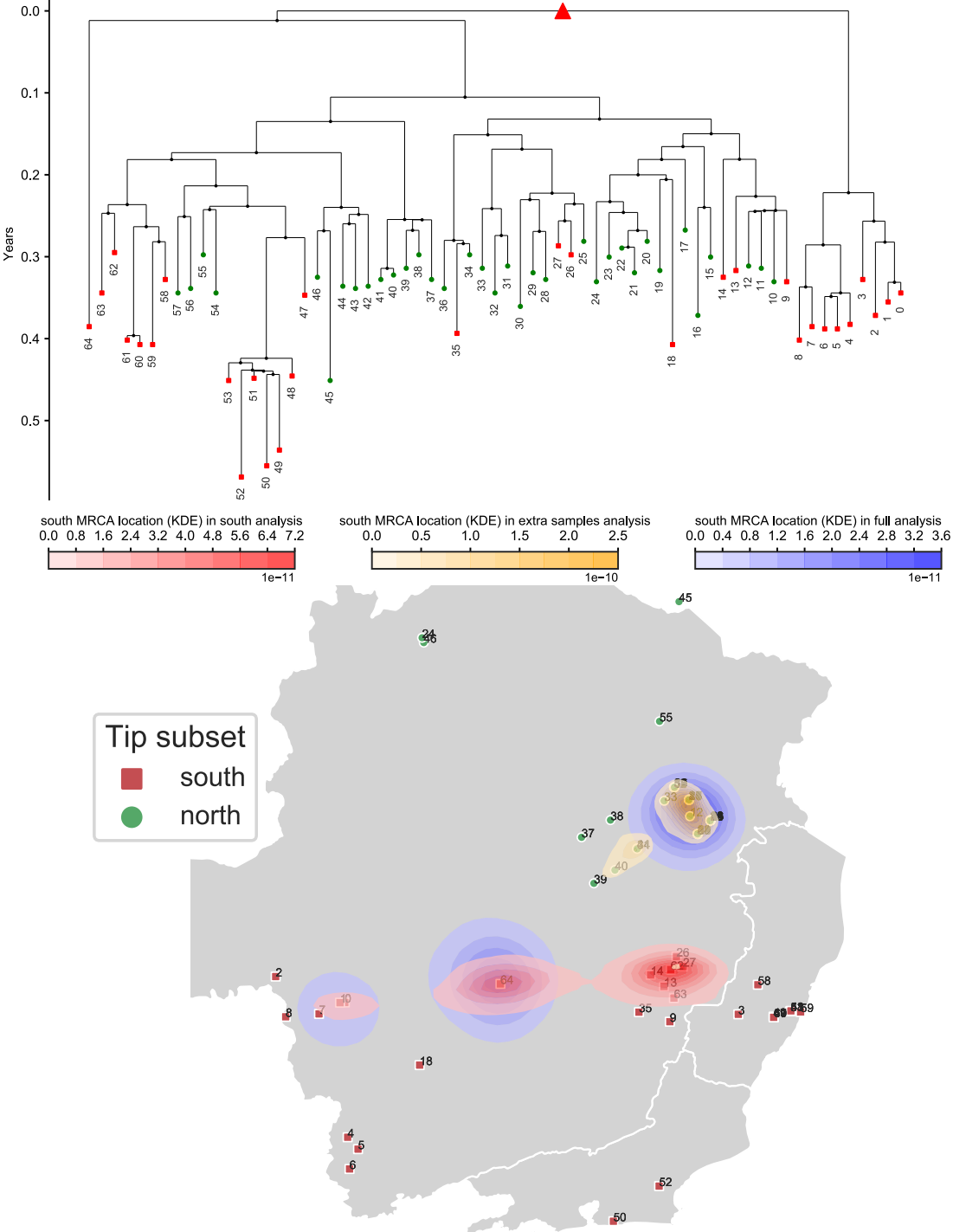

Supplement: S27 Fig — Complementarily to S26 Fig, we consider the effects or restricting an analysis to a southern geographical subsample of the YFV dataset. We compare the results of BMP analysis using all the data from [46] versus using only the southern samples (latitude below −19.0, red squares in the phylogeny and on the map). On top is the maximum clade credibility phylogeny inferred from analysing the whole dataset. On the map (bottom) we show the location of the samples and the inferred location of the most recent common ancestor (red triangle in the phylogeny). The three colored areas on the map show the inferred posterior distribution (kernel density estimate) of the root location from three analyses: using only the southern samples (red area), using all samples (blue area) or using only the southern samples but adding the northern ones as sequence-free samples (orange areas). Due to difficulties in convergence, and following results from the analysis with all samples, in the analysis with sequence-free samples we added a normal distribution prior over root height with mean 0.7 and standard deviation 0.25. (PDF) [file pcbi.1008561.s028.pdf]

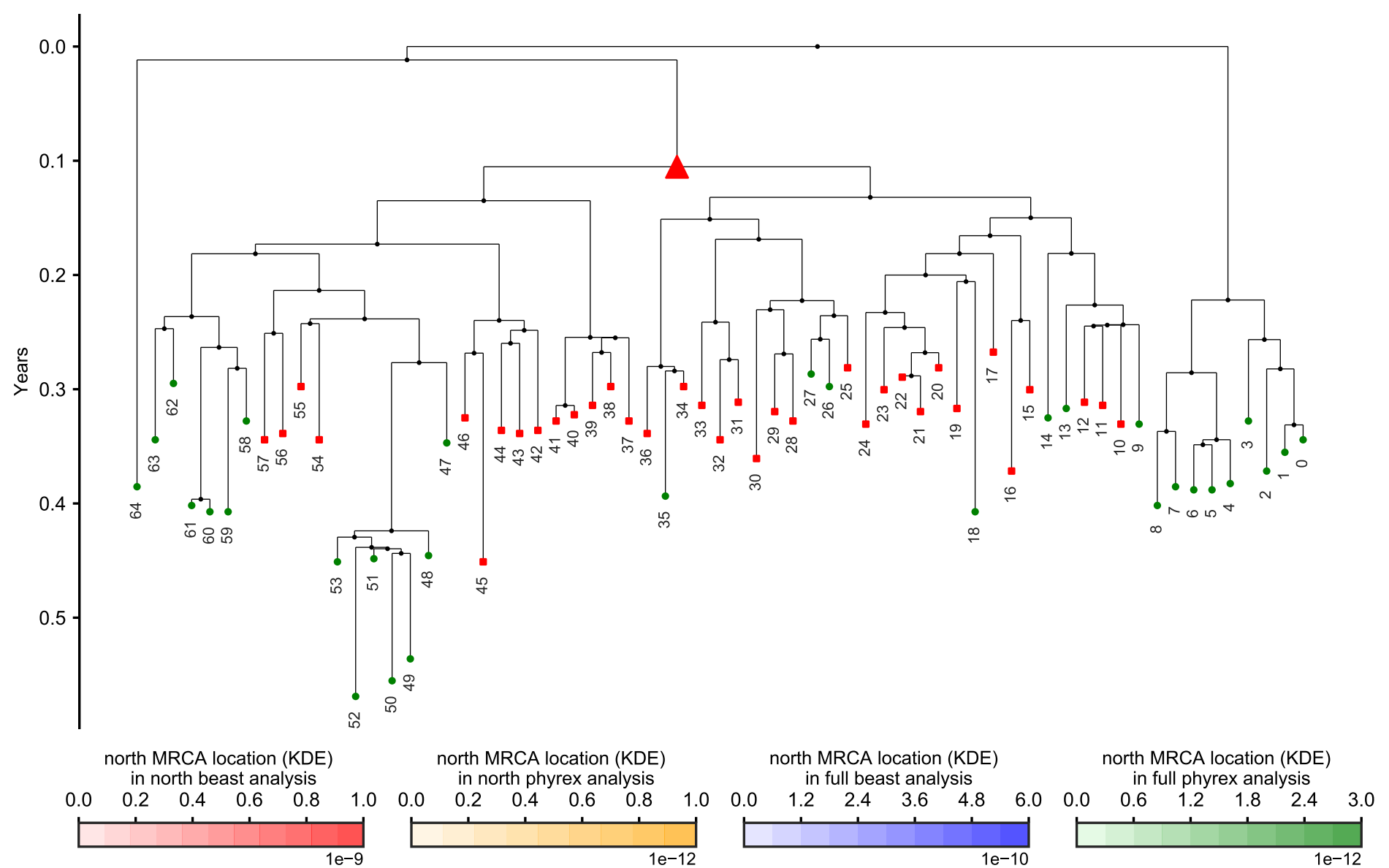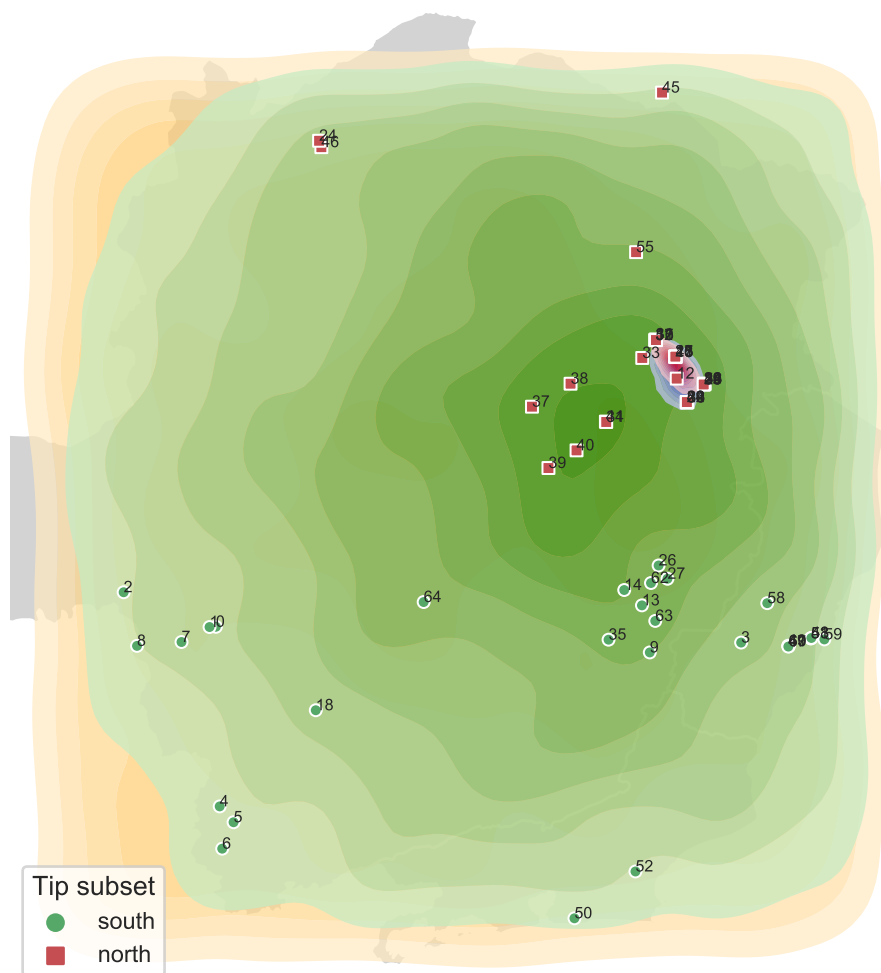

Supplement: S28 Fig — Similarly to S26 Fig, we consider the effects or restricting an analysis to a northern geographical subsample of the YFV dataset, and we compare PhyREX and BEAST inference. The colors on the map show the posterior distribution of the location of the considered ancestor for the analysis with BEAST and northern samples (red), PhyREX and northern samples (orange), BEAST and all samples (blue), and PhyREX and all samples (green). In PhyREX we defined a rectangular space with latitude interval [−23, −15] and longitude interval [−48, −40]. Posterior distribution of the diffusion rate inferred by PhyREX has a mean of 537.0 (95% HPD interval [56.8, 1030.3]) km/year in the full dataset and of 1132.2 ([68.4, 2373.6]) km/year with only northern samples. (PDF) [file pcbi.1008561.s029.pdf]

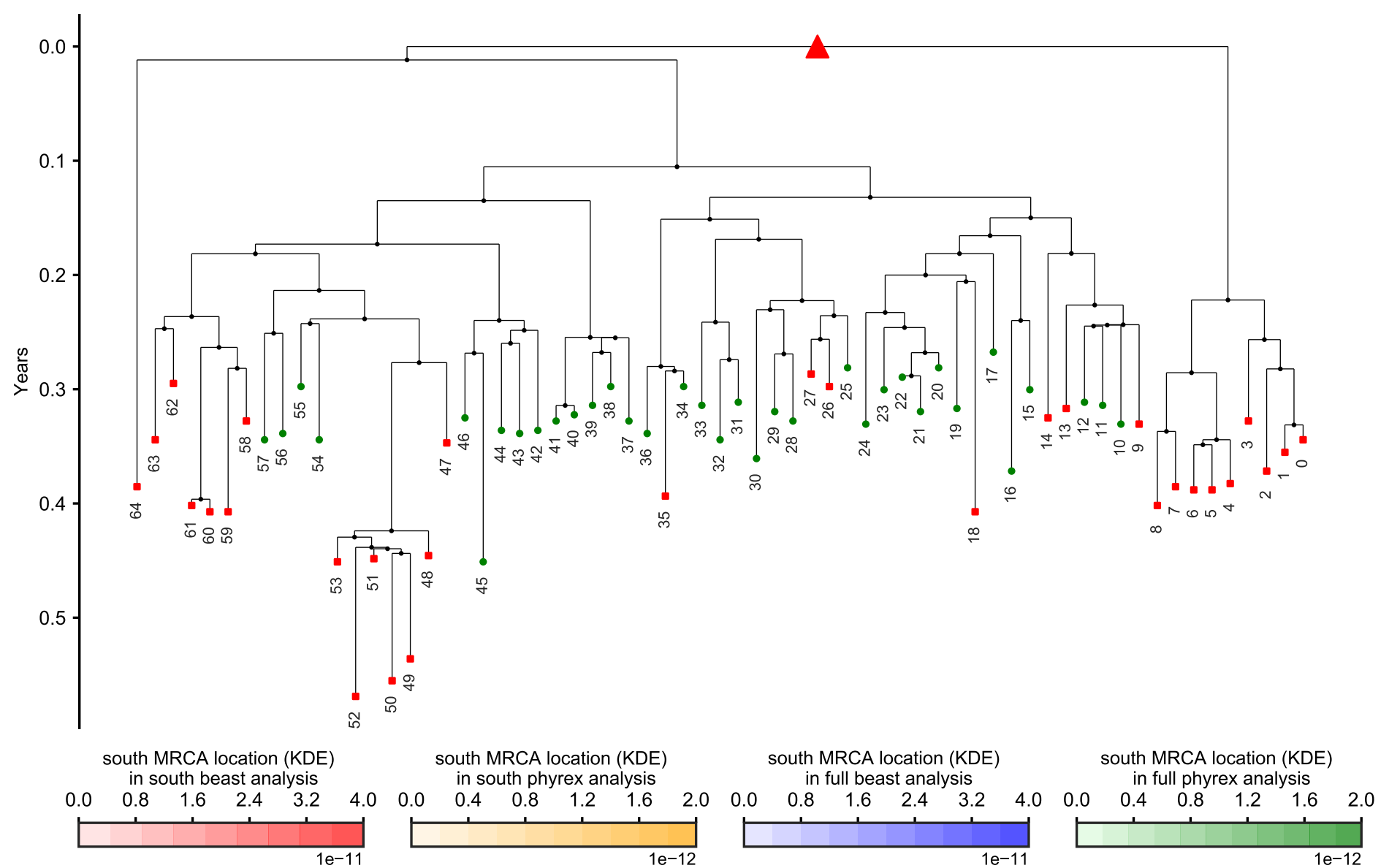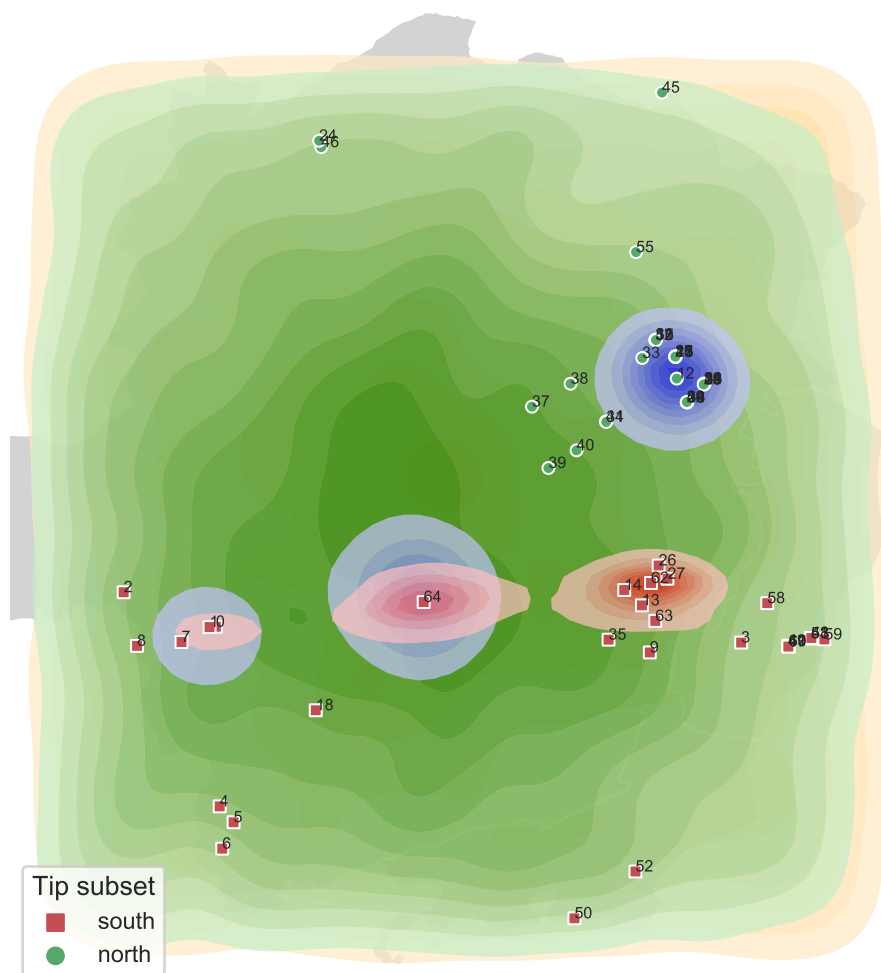

Supplement: S29 Fig — Similarly to S27 Fig, we consider the effects or restricting an analysis to a southern geographical subsample of the YFV dataset, and we compare PhyREX and BEAST inference. The colors on the map show the posterior distribution of the location of the considered ancestor for the analysis with BEAST and southern samples (red), PhyREX and southern samples (orange), BEAST and all samples (blue), and PhyREX and all samples (green). In PhyREX we defined a rectangular space with latitude interval [−23, −15] and longitude interval [−48, −40]. Posterior distribution of the diffusion rate inferred by PhyREX has a mean of 537.0 (95% HPD interval [56.8, 1030.3]) km/year in the full dataset and of 825.6 ([66.6, 1562.5]) km/year with only southern samples. (PDF) [file pcbi.1008561.s030.pdf]

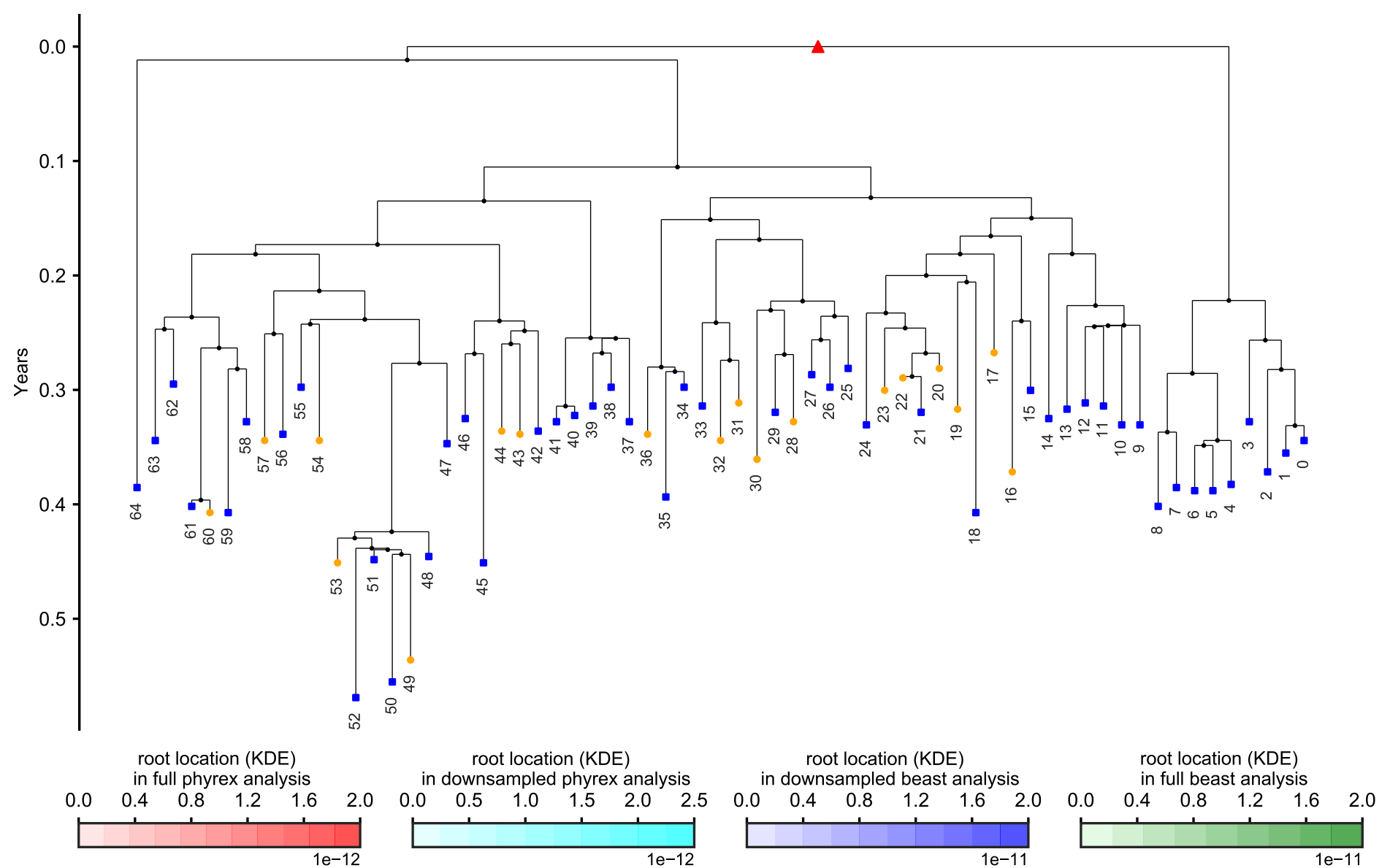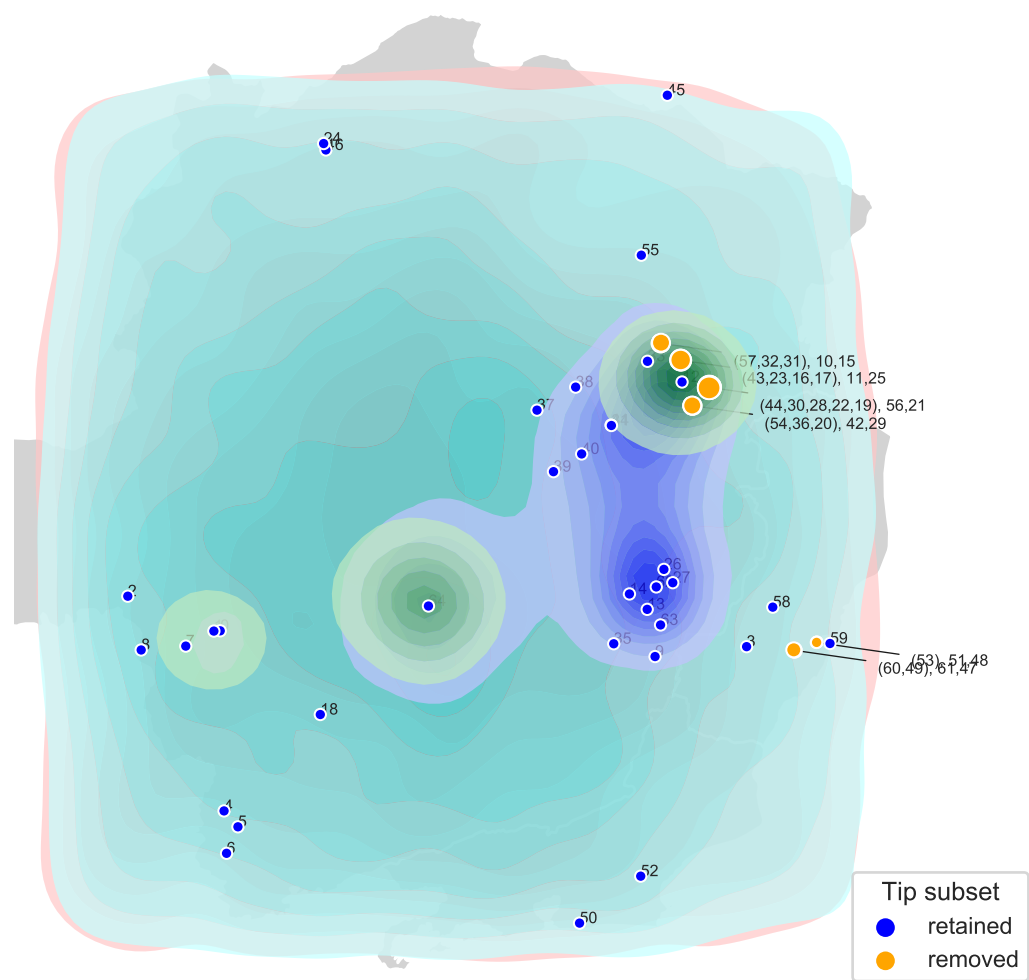

Supplement: S30 Fig — Here we compare the results of using all the YFV dataset versus downsampling each location up to allowing a maximum of two samples per location, similarly to Fig 6. Here we also compare BEAST and PhyREX inference. The green area on the map shows the inferred posterior distribution (kernel density estimate) of root location in the full analysis in BEAST, while red is the same for PhyREX. The blue area shows the posterior distribution of root location in the downsampled analysis in BEAST, while cyan is the same for PhyREX. In PhyREX we defined a rectangular space with latitude interval [−23, −15] and longitude interval [−48, −40]. Posterior distribution of the diffusion rate inferred by PhyREX in the sownsampled analysis has a mean of 537.1 (95% HPD interval [60.8, 1049.4]) km/year. (PDF) [file pcbi.1008561.s031.pdf]
