# Supplementary material for: Sampling bias and model choice in continuous phylogeography: Getting lost on a random walk
Source: PLoS Comput Biol. 2021 Jan 6;17(1):e1008561. doi: 10.1371/journal.pcbi.1008561 (PMC7815209; doi:10.1371/journal.pcbi.1008561)
Supplement: Attachment — Submitted filename: responseToDecisionLetter.pdf [file pcbi.1008561.s032.pdf]

Sampling bias and model choice in continuous phylogeography: getting lost on a random walk

Response to Decision Letter

We thank the Editor and the Reviewers for the comments to the manuscript. Please find below in italic font our responses to the Reviewer's comments. We also include a version of the manuscript with important changes marked in green.

Reviewer 1:

In their paper, the authors present a timely and important study into the effects of sampling bias on two popular continuous phylogeography models. I found the topic compelling, and the paper to be generally well written. As such I have no major objection to its being published immediately as it stands. That said, I do have several small comments/questions for the authors that they may wish to address/clarify in order to improve the submission.

Answer: *We thank the Reviewer for the comments and suggestion, which are addressed below and in the new version of the manuscript.*

1. In lines 316-318 on page 9, the authors state that the inclusion of unsequenced samples doesn't completely eliminate the bias due to nonrandom sampling, saying that to achieve this one would "probably" have to include enough additional samples to make the total distribution of samples even. How difficult would it be to show that this does actually occur, even for a small example?

Answer: *We have now added additional simulations in a scenario of one-sided sampling bias, where we simulate only 100 cases and we sampled 20 of them in a biased way. Then, we add 20 sequence-free samples at the time, until we have all 100 simulated samples, but only the 20 biased ones have sequences. The result is that we don't need to add all sequence-free samples to correct inference bias. Adding 20 samples removes most of the biases, and adding 40 leaves no clear bias in the inference, so adding more than*

40 brings no clear benefit but carries considerable additional computational demand. We have now added these analyses and results to the manuscript.

2. I'm slightly worried about the convergence criterion for the PhyREX analyses detailed on lines 334-336 on page 10. These lines do not exclude the possibility that analyses with arbitrarily low ESS values have been included in the analysis. Maybe a threshold ESS could be instigated, below which analyses are excluded? Although of course one needs to be careful there too, as such thresholds can potentially bias results too. Otherwise, would it be possible simply to run the low-ESS analyses for another week or two? I'm not really concerned that the inference results are biased due to lack of convergence, but being able definitively say that all analyses were run to some minimum ESS would remove any shred of doubt.

Answer: *Achieving convergence with PhyREX was indeed one of the most challenging parts of this work, and in the end it was necessary to run the software for a week on each replicate (given 600 replicates, that's* > 10 *years CPU time) and in some of the replicates this was not sufficient (57 out of 600 replicates had* $ESS < 100$ *for at least one of the parameters). We have now rephrased this part to clarify this, and we have added plots where these 57 runs were excluded, noting however that this is only a partial solution, and we discuss that, in future applications, possible convergence issues with the SLFV should be taken into account. The non-converged runs did not seem to show any specific trends in terms of the parameters that we consider here, and removing them from the plots did not noticeably affect any of the patterns observed.*

3. On lines 354-356 on page 10, the authors say that using a prior to limit the size of the ΛFV radius parameter improves the agreement between the inferred diffusion rate under this model and the true value, as simulated under the BMP. It's intuitively clear why the bias is present and why this trick helps, but I wonder whether another approach might be to define some kind of (approximate) effective diffusion rate on the ΛFV side which would be more directly comparable to the diffusion rate on the BMP side. The reason I'm suggesting this is that the existing "bias" seems more just a result of failing to compare apples with apples.

Answer: *Indeed, this is an important point: there is no single parameter in the ΛFV that describes diffusion rate, unlike in the BMP model. However, other authors have previously described a formula that well represents such diffusion rates in most scenarios. We have also introduced 3 additional such measures. The original formula seems the one to perform best, at least when used on ΛFV simulations where model misspecification is minimal. All these approaches are described in the Supplementary section "Estimates of diffusion rate* σ^2*". However, these issues with estimating the diffusion rate with the ΛFV model in BMP simulations don't seem related to defining a diffusion rate, but rather to the different expectations of the models in terms of past history, with the ΛFV model expecting much more migration within the defined space before lineages find a most recent common ancestor; under the BMP, on the other hand, lineages are expected to spread out from one central source, and so the total amount of migration expected (for equal sampling locations and diffusion rate) is far less. Another way to think of the difference between these two models is by considering the fact that the ΛFV happens within a limited space, while the BMP in an infinite one. Constraining the BMP within finite boundaries, and forcing the root to be relatively old with some appropriate prior, would probably make the two model dynamics more similar - although the issue of the effects of sampling biases would still remain.*

4. On lines 358-359 on page 10, the authors describe their ΛFV simulation study setup. It's not clear to me why they chose to simulate trees under the slightly different ΛFV implementation available in discsim, as opposed to precisely the model that the inference is done under. This seems like a needless complication of the study method.

Answer: *It is true that we could have designed our own simulators, but that would have meant for us to invest a lot more time and effort in programming an efficient simulator and testing it. Given that such a simulator already existed, we opted for using discsim, even though the model simulated does not match completely the one used for inference. The most important difference between the two ΛFV models seems to be the rectangular space in PhyREX versus the torus of discsim. To account for this discrepancy, we only consider in discsim samples collected from the centre of the space, so as to reduce as much as possible the impact of the topology of the space on the simulated patterns.*

Typographical errors: 1. Line 18, "include parameter" → "include parameters" 2. Line 86, "top node" could be ambiguous 3. Line 104, "nominator" → "numerator" 4. Lines 466-468 appear to be a broken sentence.

Answer: *We thank the Reviewer, these typos have now been fixed.*

Reviewer: 2

This a timely and mostly straightforward paper. I found a few issues that need clarification, and think more realistic sampling bias scenarios would greatly improve its impact, but as is I think it's a useful and somewhat disconcerting paper for the utility of phylogeographic models in estimating epidemiological parameters in outbreaks. If anything I think the authors are too positive about the performance of these methods given nonrandom sampling. Essentially, the authors summarize two continuous phylogeography models and compare their robustness to biased sampling when attempting to infer the origin of an outbreak and its dispersal rate. Given the current pandemic this is an important area of research. I found the headline findings not particularly surprising - BMP is badly affected by biased sampling, spatial-lambda-fleming-viot (SLFV for the remainder of this review) less so - and the suggestion to use sequence-free samples in BMP is well described and useful. However I was surprised how poorly the SLFV model performed, think the bias scenarios tested are too extreme, and would like to see a little more discussion of what sampling bias looks like in real data. Here I'll give some broader suggestions for improving the paper, but I don't think they're necessarily required for publication - just that they could improve it (or possibly could help guide future work).

Answer: *We thank the Reviewer for the comments and for the suggestions. Each issue is now addressed below in detail and in the new version of the manuscript.*

1. I think the authors overlook a significant issue when claiming the SLFV is not affected by sampling bias: the definition of the landscape boundaries required for inference under the SLFV will itself be shaped by the spatial distribution of samples in realistic settings.

Answer: *We think that the Reviewer is right here: in some cases the researcher might not be aware of the presence in certain regions of the organism being studied. In other cases, the researcher might instead be aware that the given samples are spatially biased and might correct for this by specifying the correct SLFV space. We have now clarified this in the Conclusion section.*

2. This is connected to a second issue ? the biased sampling scenarios tested are more extreme than I expect in a real data-deficient outbreak situation (as we are currently facing). Tests in which sampling is uneven relative to population density over space rather than completely excluding much of the map would be more relevant to real-world data analysis. What we see with covid or flu is an excess of samples in rich countries and a deficit in poor countries, relative to local viral populations, and also a severe local clustering within countries as a few hospitals do most of the sequencing. I suspect this has quite different effects on these analyses than the more extreme forms of sampling bias tested here. These scenarios are certainly useful as hyperbolic cases, but more realistic scenarios would be more impactful. However I think that is beyond the scope of a revision to the current manuscript (though I'd love to see the authors pursue it in the future!), so at least briefly discussing the sorts of bias in real-world data would be useful.

Answer: *Indeed, the scenarios considered in the manuscript consisted of very strong sampling biases. We have now added simulation results and real data results in the case of more intermediate forms of sampling biases, showing that most of the inference bias is eliminated when a small part of the sampling bias is compensated for. We also note, here and in the manuscript, that the considered sampling biases are not so unrealistic as one might think: it's not uncommon for phylogeographic studies to investigate a single country or region within a continent (we give a couple of examples), and we think it's important to point out that this can lead to severe inference biases.*

3. (Line 42 - 53) It seems important here to note that the structured coalescent is a discrete population model so requires binning populations, which forces additional ad-hoc sample choices by users. In addition, though the structured coalescent is robust to uneven sampling across populations, I don't think it is at all robust to missing samples from one "population" entirely (which seems more similar to the sampling designs tested here). Note this issue of entirely missing a population also impacts SLFV through the choice of the landscape boundaries.

Answer: *Good point about ghost demes, we have now added a mention of this issue with corresponding references. Similarly for the issue of sample binning (but this time in the paragraph above which introduces continuous phylogeography vs discrete phylogeography). We also now discuss the issue of landsacape range choice in the Conclusion section, and soften the claim of robustness of the SLFV in the Introduction.*

4. In general the results of the SLFV model are, to me, surprisingly bad - given these parameter settings and empirical data, the uncertainty on root location is so broad as to provide essentially no useful information. Do the authors agree with this assessment? Is there a setting in which PhyREX does perform well? I'd like a little more clarity on this case. Perhaps the authors could discuss PhyREX's own validation data where it presumably performs reasonably well?

Answer: *We mostly agree with the assessment, but we think this is due to the fact that, given the same diffusion rate and the same sample distribution, the time to the MRCA under the SLFV tends to be longer than under the BMP (the difference is complicated by the choice of prior over the tree in the BMP however), and the root is generally more geographically dispersed under SLFV simulations than under the BMP simulations, where instead it is usually narrowly located around the centre of the sample locations. We wouldn't say this is a bad feature of the model (or an issue of the model implementation for that matter), but it surely is something that it is important to consider. In some sense this issue arises from the SLFV conditioning on the sample locations, and therefore effectively using less data than the BMP (although this is a very simplified/distorted comparison of the two models). A similar effect is seen with the structured coalescent posterior of root location usually being much more spread out than the discrete trait analysis one. Of course, our simulation settings are not the only ones possible, and while these features remain the same if we scale up or down most parameters (such as diffusion rate, mutation rate, etc), one feature of a dataset that is expected to impact this issue a lot is the heterochronicity of the samples. For example, if one of the samples collected is very close in time and phylogenetically to the root, then the uncertainty in the inferred root locations will be much smaller for both models. In SLFV simulations, having an older sample closely related to the root is less likely than in BMP simulations, but still one would expect that in an heterochronus setting the uncertainty of both models would be reduced. The reason why we didn't explore this scenario is due to the inability of current SFLV implementations to make use of sampling time data - but this is surely an aspect that should be addressed and considered in the future. We now discuss these issues in more detail in the Conclusion section.*

5. At least one figure showing PhyREX performance on empirical data should be included in the main text, since that comparison is a major focus of the paper.

Answer: *We thank the Reviewer for the suggestion, we have now moved to the main text the old supplementary Figure S15, which has now become the new Figure 5.*

Reviewer: 3

In this paper, the authors study the effect of sampling bias on two phylogeographic models: Brownian Motion and Lambda-Fleming-Viot. The simulations and two real data analyses are well-designed for illustrating the impact of sampling bias. However, I find that the scenarios considered in the paper are too extreme. For example, in the analysis of a West Nile Virus Outbreak, the sampling bias dataset include only the western-most half of the samples. A less extreme scenario such that 80% of the data points are in the west while the remaining data points spread out may be closer to a real dataset. Therefore, I suggest the authors adding these scenarios to their studies (in both simulations and data analyses).

Answer: *We thank the Reviewer for the suggestion. We have now added new simulations with one-sided sampling bias where we mix biased samples and random samples in different proportions, creating a gradient between non-biased sampling and completely biased sampling. We observed that the inference bias does not scale linearly with sampling bias, but that reducing sampling bias by one quarter removes the majority*

fo the inference bias. We also find that at intermediate level of sampling bias the diffusion rate tends to be over-estimated rather than underestimated (as happens with totally biased sampling). We also re-create intermediate levels of sampling bias within our WNV analysis, and note similar results.
